# Seasonal oyster harvesting recorded in a Late Archaic period shell ring

**DOI:** 10.1371/journal.pone.0224666

**Published:** 2019-11-20

**Authors:** Nicole R. Cannarozzi, Michal Kowalewski

**Affiliations:** Florida Museum of Natural History, University of Florida, Gainesville, Florida, United States of America; Institute for Anthropological Research, CROATIA

## Abstract

The function of Late Archaic period (5000–3000 B.P.) shell rings has been a focus of debate among archaeologists for decades. These rings have been variously interpreted as a product of seasonal feasting/ceremonial gatherings, quotidian food refuse generated by permanent dwellers, or a combination of seasonal and perennial activities. Seasonality of shell rings can be assessed by reconstructing the harvest time of oysters (*Crassostrea virginica*), the primary faunal component of shell rings. We estimated the timing of oyster harvest at St. Catherines Shell Ring (Georgia, USA) by statistical modeling of size frequency distributions of the impressed odostome (*Boonea impressa)*, a parasitic snail inadvertently gathered by Archaic peoples with its oyster host. The odostome samples from three archaeological excavation units were evaluated against resampling models based on monthly demographic data obtained for present-day populations of *Boonea impressa*. For all samples, the harvest was unlikely to start earlier than late fall and end later than late spring, indicating that shell deposits at St. Catherines Shell Ring formed seasonally with substantial harvesting activities restricted to non-summer months. For all samples, the resampling models indicated that harvesting activities likely occurred during multiple months. However, these analytical outcomes would also be expected in the case of extensively time-averaged records of short-term, non-summer harvest events. Regardless of the exact harvest duration, the results point to seasonal harvesting and suggest that Archaic populations may have opted out of consuming summer oysters to focus on other resources, avoid unpalatable food, decrease pathogen risks, or ensure sustainable harvesting.

## Introduction

Shell rings are circular or arcuate accumulations of shell, bone, artifacts, soil and other anthropogenic sediments deposited along the coasts of Florida, Georgia, South Carolina, and Mississippi [1,2]. Three interpretations dominate the discussion on shell ring use and occupation. The first hypothesis posits that shell rings represent daily food refuse discarded behind or underneath households constructed on top or inside shell ring walls [3,4]. Under this scenario, shell rings represent gradually accumulated deposits, representative of the domestic refuse of daily village life. The second hypothesis posits that shell rings represent intermittently occupied, large scale, communal feasting sites that developed in concert with other manifestations of monumentality, such as hierarchical social structures [5–7]. This hypothesis posits that ring deposits would be highly seasonal in nature reflecting intermittent use during feasting and ceremony. Under this scenario, shell rings formed by quick deposition of large amounts of shell, experienced little post-depositional crushing, and contain higher frequencies of decorated pottery than contemporaneous non-ring, shell midden sites [6–8] (see also [9] for further discussion). Finally, the third hypothesis postulates that shell rings formed as a result of both mundane and ritual practice and that site use practices may have changed over time [10]. Understanding the timing of deposition of shell ring refuse is essential to assessing these three hypotheses. Here, using a computer-intensive statistical approach, we assessed seasonality and duration of shellfish harvesting for one shell ring. Whereas the improved understanding of harvest timing cannot fully resolve the rival hypotheses regarding the origin and function of shell rings, it should augment our understanding of the seasonal dynamics of shellfish gathering and shell-ring building activities.

Oysters (*Crassostrea virginica*) are the most numerous shellfish species in nearly all shell rings, suggesting that they were a key component of the practices that contributed to ring formation [11]. Consequently, determining the season(s) of death of oysters deposited in shell rings is crucial for evaluating the timing of ring building practices and, in combination with other data types, can augment our understanding of ring formation and use over time. Currently, stable isotope methods are the most common strategy for assessing oyster harvest time. However, the cost necessary for this type of analysis can limit sample size. Additionally, the use of shell morphology (e.g., growth rings) to assess ontogenetic age and seasonal growth patterns in oysters is affected by interpretative uncertainties [12]. Consequently, combining isotope approaches with other independent strategies can augment our ability to estimate the timing of oyster harvests.

Russo [12] developed an indirect method of estimating oyster season of capture by focusing on the odostome snail (*Boonea impressa)*, a pyramidellid endoparasitic gastropod that preferentially feeds on the soft tissue of oysters and is widely distributed along the coasts of the Gulf of Mexico and Atlantic [13]. *B*. *impressa* lives for approximately one year and spawns principally in late spring [14,15]. The principal cohort grows in length throughout the year until death and is then replaced by the new cohort [15]. Previous studies on contemporary odostome populations from North Carolina and Texas [15] report comparable reproduction and growth patterns with the majority of juveniles occurring in the mid-summer months. The annual life span and invariant growth across populations from different regions and habitats [16,17] may make odostomes a suitable candidate for estimating the timing and duration of oyster harvest (but see [18]). Whereas this strategy was employed successfully in interpreting season of oyster harvest for archaeological sites in Florida and Georgia [12,19,20], its potential to provide detailed quantitative estimates of not only approximate harvest time, but also harvest duration, has not been exploited. Using R [21], we employ computer-intensive statistical modeling to assess demography of archaeological samples of *B*. *impressa* in the quantitative reference framework provided by monthly census samples of recent conspecific populations collected from the same location. Using this strategy, we report high-resolution, quantitative estimates of both the duration and seasonal timing of oyster harvesting by Archaic period peoples. These quantitative estimates of the temporal structure of shell accumulations provide a statistical approach toward evaluating the formation of the Archaic shell ring structures. We focus here on an extensively studied exemplar shell ring (St. Catherines Shell Ring, Georgia, USA).

Due to constraints of sample size on statistical analysis, sets of individual archaeological samples have been pooled and are therefore analytically time averaged. The pooling of samples is not ideal for season of harvest determination over smaller time scales however the challenge lies in assuring statistically adequate numbers of snail specimens at appropriate resolutions. Given this limitation, there are three hypotheses that can be postulated for extensively time-averaged samples:

1. The harvest occurs randomly or continuously throughout the year–in this case time-averaged archaeological samples will correctly fail to indicate seasonal harvest.
2. The harvest occurs non-randomly through the year (seasonally), but this non-random pattern changes over decades or centuries represented by each low resolution sample–in this case time-averaged archaeological samples will incorrectly indicate continuous harvest and fail to detect the fact that the harvest was actually seasonal in any given year. However, the comparative analyses of multiple samples would likely point to significant among-sample differences correctly indicating that harvesting patterns varied through time.
3. The harvest occurs non-randomly through the year (seasonally) and this pattern persists through the multiple decades/centuries represented by each sample–in this case the time-averaged archaeological samples should correctly support non-random (seasonal) harvesting as well as the long-term (multi-centennial) persistence of that seasonal harvesting pattern. However, substantial, potentially multi-centennial time-averaging could result in making short-term (1-month or less) harvesting appear more prolonged. That is, if harvest occurred seasonally once a year (e.g., a few winter days but with exact timing varying by weeks or months from year to year), time-averaging could produce the record that suggests multiple months of harvest. Moreover, because not only the harvest time, but also the spawning time and growth rate of *Boonea impressa* could vary subtly from year to year depending on environmental fluctuations, a multi-monthly signature could emerge even if harvesting occurred briefly and always in the same month. Thus, in the case of the third hypotheses, seasonality can be detected but the exact duration of harvest suggested by the data may be difficult to estimate precisely.

Note that the above hypotheses indicate that the seasonal signal in time-averaged samples is only possible to detect when consistent, seasonal harvesting persisted through time (the third hypothesis). The predictions of hypotheses 1 and 2 cannot be readily resolved using the proposed approach due to inadequate temporal resolution of pooled sample sets.

In most cases, it is not appropriate to pool zooarchaeological samples across time and space because combining samples hampers our ability to detect variations in seasonal deposition of discrete or synchronous deposits. However, in the specific case of the hypotheses evaluated in this study, pooling of samples is a conservative approach. This is because, in pooled samples analytically time-averaged over decades or centuries, seasonal patterns are detectable only if harvest seasonality is persistent through time and harvest seasons are highly consistent across years. Additionally, column samples may not be representative of a deposit or an entire site but, when combined can contribute to our overall understanding of site composition and deposition [22]. We utilize this approach here because it is a logistic prerequisite for acquiring adequate sample size and because, more importantly, pooling represents a maximally conservative approach for detecting harvest seasonality. The approach proposed here is reproducible and the analytical and sampling methodology are transferrable to other archaeological sites and site types as long as the tested hypotheses focus on long-term processes such as multi-centennial persistence of seasonal harvest.

## Materials and methods

### Study site

**Fig 1:**
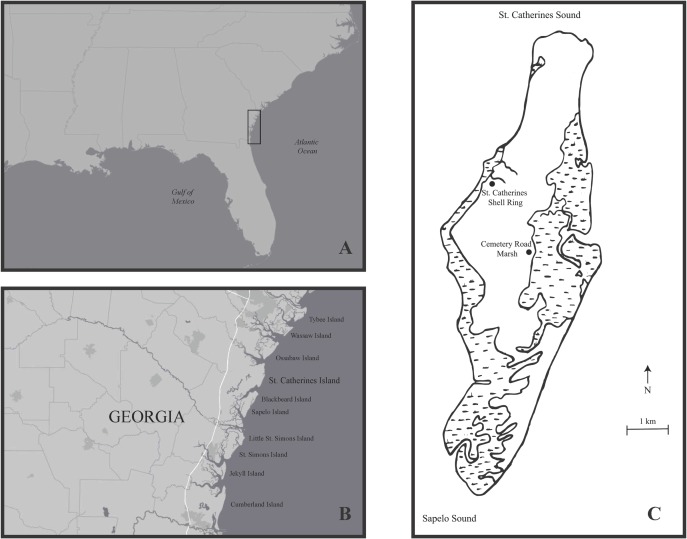
Location of St. Catherines Island. (A-B) Location of St. Catherines Island. (C) St. Catherines Shell Ring and *Boonea impressa* collection site, Cemetery Road Marsh.

St. Catherines Shell Ring, one of two contemporaneous shell rings on St. Catherines Island off the coast of Georgia, USA (Fig 1), counts among the most extensively studied shell rings in the southeast. This study site has produced large datasets of substantial interpretative value, including ceramic, lithic, geophysical, faunal and botanical data. The ring, located on the western marsh, is 70 m across, its interior spans 34 m, and consist of shell deposits that vary from 25cm to 1m in thickness [23]. The faunal and artifact composition of the ring are similar to those of other Late Archaic shell rings in the southeast [23,24]. Radiocarbon dates of shell deposits from St. Catherines Shell Ring date to 2230–2030 cal B.C., indicating that ring deposition took place over a period of ~200 years [23]. All calibrated radiocarbon dates available for the study site have been reported previously ([23]; Table 3.1 therein).

### Sampling recent and archaeological populations of *Boonea impressa*

The described study did not involve endangered or protected species and no permits were required. This study combines two distinct types of data: (1) Recent samples of live-collected specimens that were acquired monthly and (2) archaeological samples from shell layers preserved within the St. Catherines Island Shell Ring structure. Recent and archaeological samples are conspecific and geographically sympatric.

To obtain a modern comparative proxy, live specimens of *B*. *impressa*, live oyster clumps (~30 oysters per clump) were randomly collected monthly from July 2006 to June 2008 from Cemetery Road Marsh, an intertidal oyster reef located on the eastern side of St. Catherines Island (Fig 1). Odostomes are small and difficult to see because exposed intertidal oysters are covered in mud deposited from the receding high tide, so it is challenging to collect them individually in the field. Thus, oyster clumps were transported back to the Environmental Archaeology Lab at the Florida Museum of Natural History where they were soaked in water, separated into individuals, brushed and rinsed to dislodge all odostomes from their oyster host. The resulting silt and water was wet-sieved through nested geological sieves of 6.8 mm, 3.3 mm, 1.168 mm, and 250 μm to facilitate separation of odostomes from larger shell fragments, mud and other debris. Odostomes were recovered only from the 1.168 mm and 250 μm sieves and identified using low power microscopy to assure that specimens were not broken or dead at the time of collection. Because of this sampling strategy, a direct assessment of infestation burden (i.e., percentage of snail-infested oysters) is not possible. However, the fact that ~4500 specimens were recovered from 720 oysters and virtually all clumps of oysters produced sieved residuals with substantial numbers of snails indicate that infestation rates were exceedingly high and persistent seasonally. This is consistent with ecological studies from multiple regions which all suggest that *Boonea impressa* tends to be ubiquitous, infesting oysters throughout the year [25,26], with single oyster host supporting aggregates of as many as 100 parasitic snails [27]. The previous studies also suggest that the spatial distribution of snails is independent of host population density and tend to aggregate based on the presence of other snails [13]. The oyster reef within the Cemetery Road Marsh was a densely packed reef, which should have further facilitated widespread infestation by odostomes. Oyster clumps were gathered from various areas of the reef representing varying shell lengths so as not to bias collections toward older or larger individuals as well as potential differences in spatial distributions. Given its ubiquity and continued presence through the seasons, *Boonea impressa* is expected to be an effective harvest proxy.

All gastropod specimens identified as *Boonea impressa* were distinguished from congeneric species by the channeled sutures, spiral grooves and flared aperture [28]. Identifications were made using the zooarchaeology comparative collection at the Florida Museum of Natural History, University of Florida, Gainesville. Modern odostomes and archaeological collections described in this study are on loan from the Laboratory of Archaeology, University of Georgia, Athens. All specimens are housed temporarily in the Environmental Archaeology Laboratory at the Florida Museum of Natural History (Table B in S1 Appendix).

**Fig 2:**
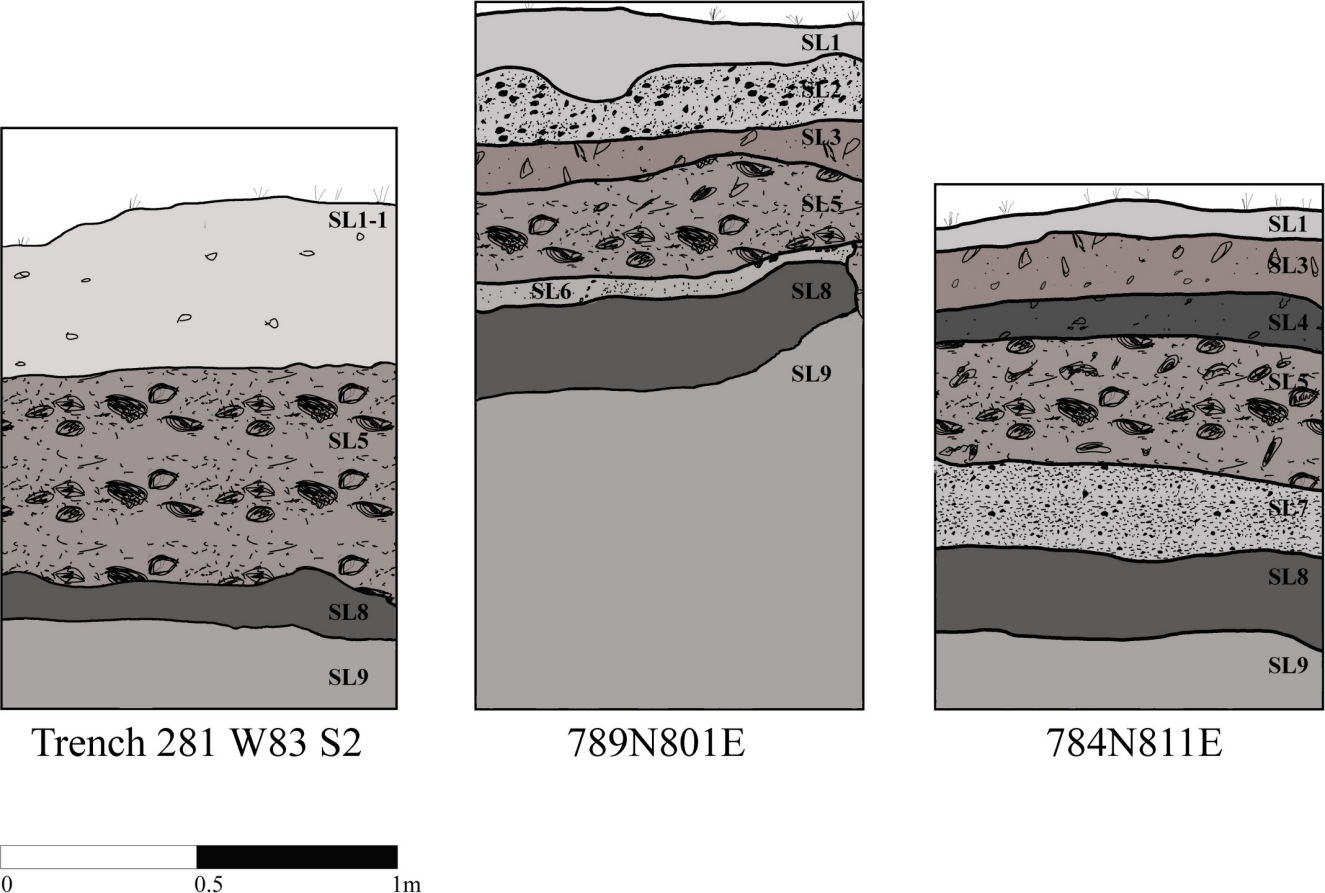
Stratigraphic profiles. Profiles detailing the stratigraphy of the three units from which column samples were excavated. SL1-1 consists of overburden from a historic trench; SL2 is a sandy loam with whole and fragmented oysters, clams, periwinkles, mussels, and bone; SL3 is a sand layer with relatively abundant, but highly fragmented, shell material; SL4 consists of sand with sparsely dispersed shell fragments; SL5 is a sandy loam soil with a dense concentration of shells consisting of whole and fragmented oysters, clams, periwinkles, mussels, and other faunas; SL6 is a light gray sand layer with highly fragmented shell material; SL7 is a sand layer with abundant but heavily fragmented shell material; SL8 is a buried A horizon, and SL9 is a variable sand layer. Modified from Sanger 2015 [26].

To obtain archaeological samples, three 25x25x10 cm column samples were excavated from three shell-rich units within the ring: Trench 281-W83S2 (281-W83S2), 784N811E, and N789E801. Trench 281-W83S2 and N789E801 extended over 1m below site datum and 784N811E extended 0.76m below site datum. The deposits in each unit are variable but the base of each unit consisted of a sand layer atop a buried A horizon upon which the ring initially formed [26]. As standard practice, the surface layers of all units were not included in the faunal analysis and were therefore not included in the subsequent analyses. Trench 281- W83S2 is located on the western side of the shell arc, along a historic agricultural boundary ditch. As a result, the upper 30 cm was disturbed and is not included in the study (Fig 2, SL1-1). The undisturbed layer below consisted primarily of a mix of whole and fragmented oysters, clams, periwinkles, crushed mussels, and bone with little soil (Fig 2, SL5). This shell-rich layer lays directly above a sand layer (Fig 2, SL9). Throughout the ring, the sand layer ranges in color from yellowish-brown to pale brown and varies in composition from sand, sandy loam or loamy sand [26].

Unit 789N801E is located in the thickest portion of the northern part of the ring. The column sample for this study was taken from the southern wall of the unit and consisted of seven stratigraphic layers. Above the sand layer (Fig 2, SL9) and A horizon (Fig 2, SL8), there is a thin layer of highly fragmented shells mixed with light gray sand (Fig 2, SL6). The stratum above is a dense accumulation of shell, including whole oysters, clams, periwinkles, mussel fragments, and bone mixed with light gray sand (Fig 2, SL5). The shell deposit is less dense in the stratum directly above, which consists of sand and mostly fragmented shells (Fig 2, SL3). The uppermost layer below the surface (Fig 2, SL1) is sandy loam (Fig 2, SL2). Here, the density of shell material increases again and is dominated by whole and fragmented oysters, clams, periwinkles, mussels, and bone (Fig 2, SL2).

Unit 784N811E is located east of 789N801E. The column sample was taken from the northern wall of the unit and consisted of seven stratigraphic layers. Directly above the buried sand layer (Fig 2, SL9) and A horizon (Fig 2, SL8), is a thin layer of highly crushed shell and sand (Fig 2, SL7) that underlies a stratum of primarily whole mollusk shells within a light gray sandy matrix (Fig 2, SL5). The overlying stratum, shared with 789N801E and Trench 281-W83S2, is a sandy loam containing whole and fragmented oysters, clams, periwinkles, mussels, and other faunas (Fig 2, SL5). The stratigraphic unit directly above is a layer of sand with sparsely dispersed and highly fragmented shell debris (Fig 2, SL4). The last stratum below the surface layer (Fig 2, SL1) is a gray sand unit with densely packed and crushed shell (Fig 2, SL3).

**Fig 3:**
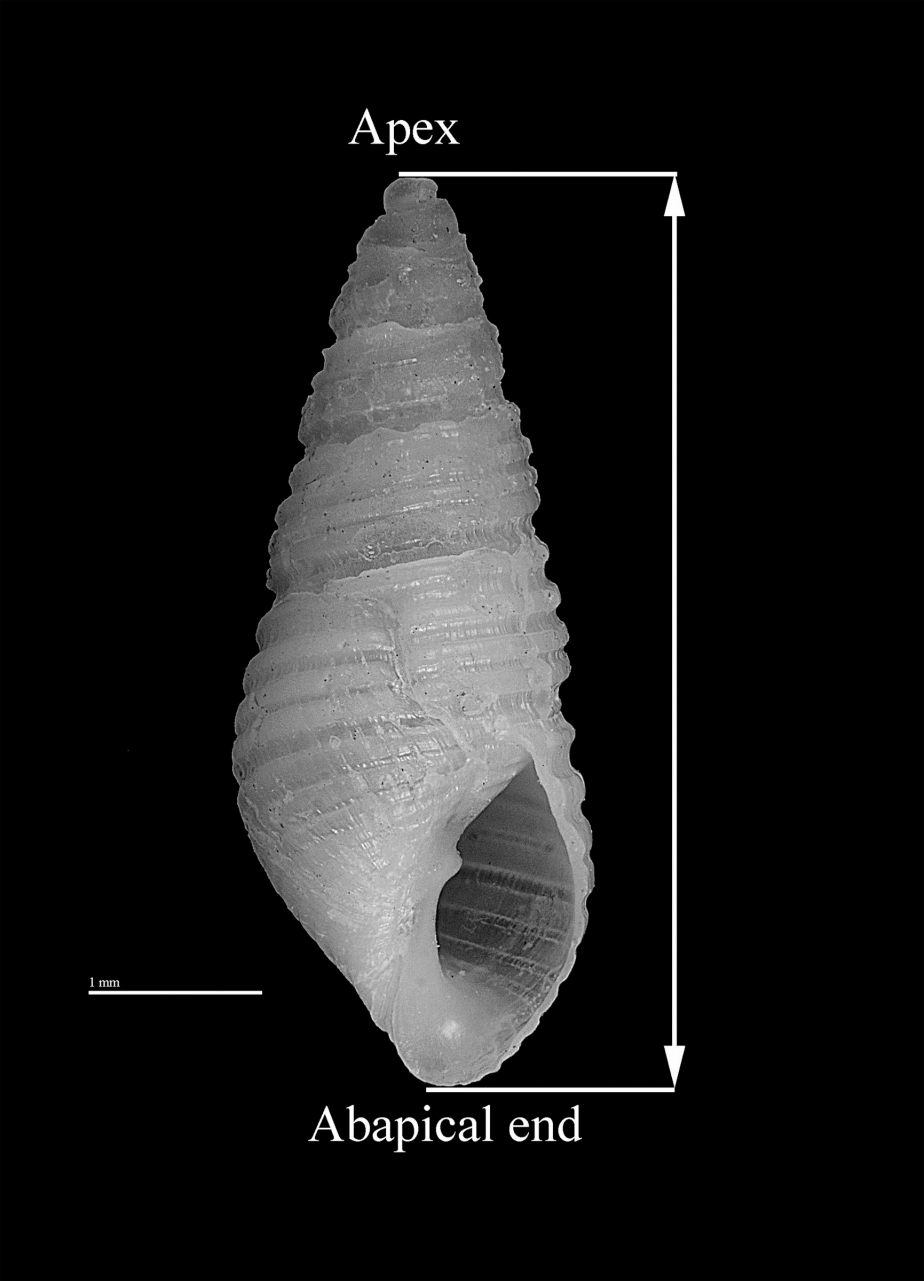
*Boonea impressa*. A specimen of the odostome gastropod *Boonea impressa*. Arrows and reference lines indicate the maximum shell dimension (shell height) measured to estimate snail body size.

Each column sample was excavated in arbitrary 10 cm levels and all material was water-screened through nested sieves of 6.35 mm, 3.18 mm and 1.59 mm gauge. The invertebrate fauna were consistent in species composition throughout the three columns, containing primarily oysters, ribbed mussel (*Geukensia demissa*), and marsh periwinkle (*Littorina irrorata*) (Table A in S1 Appendix). Odostomes were recovered and identified during zooarchaeological analysis of the invertebrate fauna. Odostomes were primarily concentrated in 1/16^th^, and rarely the 1/8, screen fractions. Each specimen was checked for breakage prior to measurement. Following Russo [12] shell height was measured from the apex to the abapical end (Fig 3) using electronic calipers with precision of +/- 0.02 mm. All live-collected specimens included the apex and abapical end and could be measured reliably.

Because individual 10 cm levels did not contain enough snails for statistical analysis, the column samples were pooled by excavation unit to obtain the three samples used in the final analyses (281-W83S2, 784N811E, and 789N801E). Note that these three vertically aggregated samples span the entire thickness of the ring, and thus, each sample likely represents a comparable record representing ~200 years of accumulation. Thus, samples are time averaged over multi-centennial time-scales. Alternatively, samples can be pooled horizontally across the three excavation units to generate a sequential time-series of samples representing shorter time intervals. To ensure adequate sample size, we have grouped subsamples into three samples representing the same elevation ranges (bottom = elevation below 2.4 m, middle = 2.4–2.7 m elevation, and top = elevation above 2.7 m). Elevation at the site was determined using an absolute depth system in which a laser level was used to measure actual elevation in relation to an arbitrary 2-meter site datum. However, the horizontal samples are also likely to be substantially time-averaged because the elevation alone does not guarantee precise temporal correlation across different parts of the shell ring and age mixing may be substantial even within individual (10cm thick) column samples. The horizontally pooled samples represent less time-averaged samples than vertical samples, but are more extensively averaged spatially. These three horizontal and three vertical samples were compared to each other to assess both lateral and temporal differences in size-frequency distributions of archaeological samples of *B*. *impressa* (S1 Appendix). The vertical samples are referred to as 281-W83S2, 784N811E, and 789N801E, where the horizontal samples are referred to as 2.1, 2.4, and 2.7.

Because the American Museum of Natural History’s archaeological program used 1.6 mm screen as the finest mesh size for St. Catherines Shell Ring and the recent population samples were recovered using 0.25 mm screen, the comparisons of size frequency distribution (SFD) data may be invalidated by methodological discrepancies. The smallest specimens observed in recent samples were 0.34 mm in size and although specimens below 0.5 mm were rare (<0.5%), 33% of specimens were below 1.6 mm (mesh size used in archaeological sampling). However, the size frequency distribution of archaeological specimens indicates that absence of small specimens is not due to the application of coarser mesh size. The smallest specimen is 2.16 mm and specimens below 2.5 mm are exceedingly rare (<0.4%) and even those <3mm represent only 3.3% of specimens. This indicates that specimens below 3mm are nearly absent in the archaeological samples and thus the use of 1.6 mm mesh size was inconsequential. The presence of small terrestrial gastropod species suggests that the absence of small odostome specimens is not likely due to preservation bias. Generally, the shells of terrestrial gastropods are thinner than those of marine gastropods [29] and are less likely to survive over time. Terrestrial gastropod species with average shell length measurements of 1.5 mm including the minute gem (*Hawaiia minscula*), slim snaggletooth (*Gastrocopta pellucida*) and bottleneck snaggletooth (*Gastrocopta contracta*) in the sample indicate that conditions were amenable to the preservation of small odostome specimens. Nevertheless, to further evaluate this methodological bias, all analyses presented below were also repeated with all recent specimens <1.6 mm removed from the analysis. As documented below, these supplementary analyses were consistent with those performed on untrimmed data.

### Size-frequency distributions

The size-frequency distributions of recent (live-collected) populations of *B*. *impressa* (R-SFDs) were initially analyzed by month (24 monthly live samples spanning from July 2006 through June 2008). Because samples collected in the same month during successive years (e.g., June 2007 vs. June 2008) yielded consistent size-frequency distributions (see below), the data were pooled across the years to increase the sample size. Consequently, the comparative analyses were based on 12 monthly R-SFDs, with data pooled across multiple years.

Archaeological odostomes could have been collected over one month, multiple consecutive months, multiple non-consecutive months, or continuously throughout the year. Consequently, both specific harvest month(s) and harvest duration (number of months) need to be considered. We do not suggest that this method alone can determine the specific months in which oyster harvest took place (see interpretative caveats of Hypothesis 3 discussed above). Rather, the estimates with a specific start and end month is viewed here as a first-order analytical outcome that should not be interpreted directly in terms of duration of harvest. Instead, these estimates serve as a constraining tool when interpreting the seasonality of oyster harvest. The monthly R-SFDs allow for deriving 144 R-SFDs (12 possible harvest start months and 12 possible durations from 1 month to 12 months). That is, there are 12 single month R-SFDs, 12 two-month R-SFDs (i.e., data pooled for two consecutive months starting with January + February and ending with December + January), 12 three-month R-SFDs, and so on, up to 12 R-SFDs for twelve consecutive months. Because 12-month sets are identical regardless of the start month (e.g., pooling data from June through May and from March through February produces the same dataset), there are in fact only 133 unique R-SFDs. The R-SFDs were numbered consecutively from 1 to 133. For example, R-SFD #1 is (January), R-SFD #34 (October + November +December), R-SFD #59 (November + December + January + February + March), and so on (see S1 Appendix for details). These R-SFDs represent 133 size-frequency distributions (133 R-SFDs) that provide empirical estimates of size-frequency distributions of *B*. *impressa* expected for harvests that started in a given month and lasted a given number of months. Note that the approach based solely on 133 R-SFDs disregards more complex scenarios that include harvesting with gaps (e.g., January + April + May). Considering all possible permutations is computationally expensive in simulations implemented here. However, a small set of discontinuous models were included *post hoc* as supplementary analyses to evaluate if a multi-month (but discontinuous) harvesting may explain the observed results.

### Statistical modeling

All simulations and figures were generated using custom scripts (S1 Appendix; also available at https://github.com/MJKowalewski/Boonea) written in R [21].

A simple approach to estimate harvest time of a given archaeological sample is to compare that sample to each of the 133 reference R-SFDs and identify the best distribution match. To evaluate the harvest time for archaeological samples, each archaeological SFD (A-SFD) can be compared against R-SFDs in a series of pairwise comparisons. The pairwise difference between a given A-SFD and a given R-SFD can be measured using Kolmogorov-Smirnov D (a maximum difference between two relative cumulative frequency distributions). D ranges from 0 to 1, where 0 indicates identical distributions and 1 represents completely non-overlapping distributions. The pairwise match with the lowest D value identifies the R-SFD that is most congruent with a given archaeological sample. However, this approach does not allow for robust assessment of statistical uncertainty of the resulting outcome and assumes incorrectly that 133 R-SFDs are error-free reference standards. Consequently, we propose here a modeling approach to provide a more rigorous assessment of harvest time (see S1 Appendix for details).

The analytical strategy proposed here is based on computer-intensive resampling that represents a machine-learning strategy where known distributions (133 R-SFDs) are used to develop predictive models (training sets) against which the evaluated datasets (archaeological samples in this case) are assessed numerically. Specifically, 133 R-SFDs represent reference month sets (known harvest times) that can be used to develop 133 predictive null models. Each of those null models is developed by iterative comparisons of bootstrap samples of a given R-SFD to all 133 R-SFDs using a sample size *n* (number of specimens) of the archaeological sample. This simulation produces 133 null models, one for each R-SFD. The analogous model derived for the evaluated archaeological sample is then compared against each of the 133 null models. The null models most similar to the archaeological sample model provide best estimates of the harvest time. The similarity can be measured using either presence-absence (e.g., Jaccard, Sorensen) or abundance-based (e.g., Bray-Curtis) similarity indices. While the proposed strategy is not a Maximum Likelihood Estimation (MLE) (the method maximizes non-parametric similarity rather than the actual likelihood), it is analogous to MLE in that it aims to select the most likely of many null models. In addition, because all models have the same parameters and are based on the same sample size (*n* of the evaluated archaeological sample) the information criteria adjustments [Akaike information criterion (AIC) or Bayesian information criterion (BIC)] are unnecessary [for more details about the resampling protocol see supplementary material, S1 Appendix, Methods].

All models are based on 2000 bootstrap replicate samples carried out separately for each archaeological sample. Repeated runs indicate that estimates are reasonably stable when 2000 replicates are used. Because 133 models each involving 133 comparisons are developed for each sample, an analysis of one archaeological sample requires (133*133*2000 iterations = ~35 million iterations) and for this reason generating more than 2000 bootstrap replicates was deemed impractical. On a reasonably powerful personal laptop (1.9 GHz, 16GB RAM, 64-bit system), and without employing parallel (multi-core) processing, a single simulation (2000 iterations) for the specific datasets used here requires approximately 1 hour.

The custom source codes, the raw datasets, and simulation outputs needed to replicate all analyses and analytical figures presented in this paper are freely accessible on GitHub at https://github.com/MJKowalewski/Boonea. The collection accession numbers (catalogue numbers) and geographic and stratigraphic information for archaeological and modern specimen lots used in this study are provided in an appendix (Table B in S1 Appendix) and are permanently accessible online at https://github.com/MJKowalewski/Boonea.

## Results

**Fig 4:**
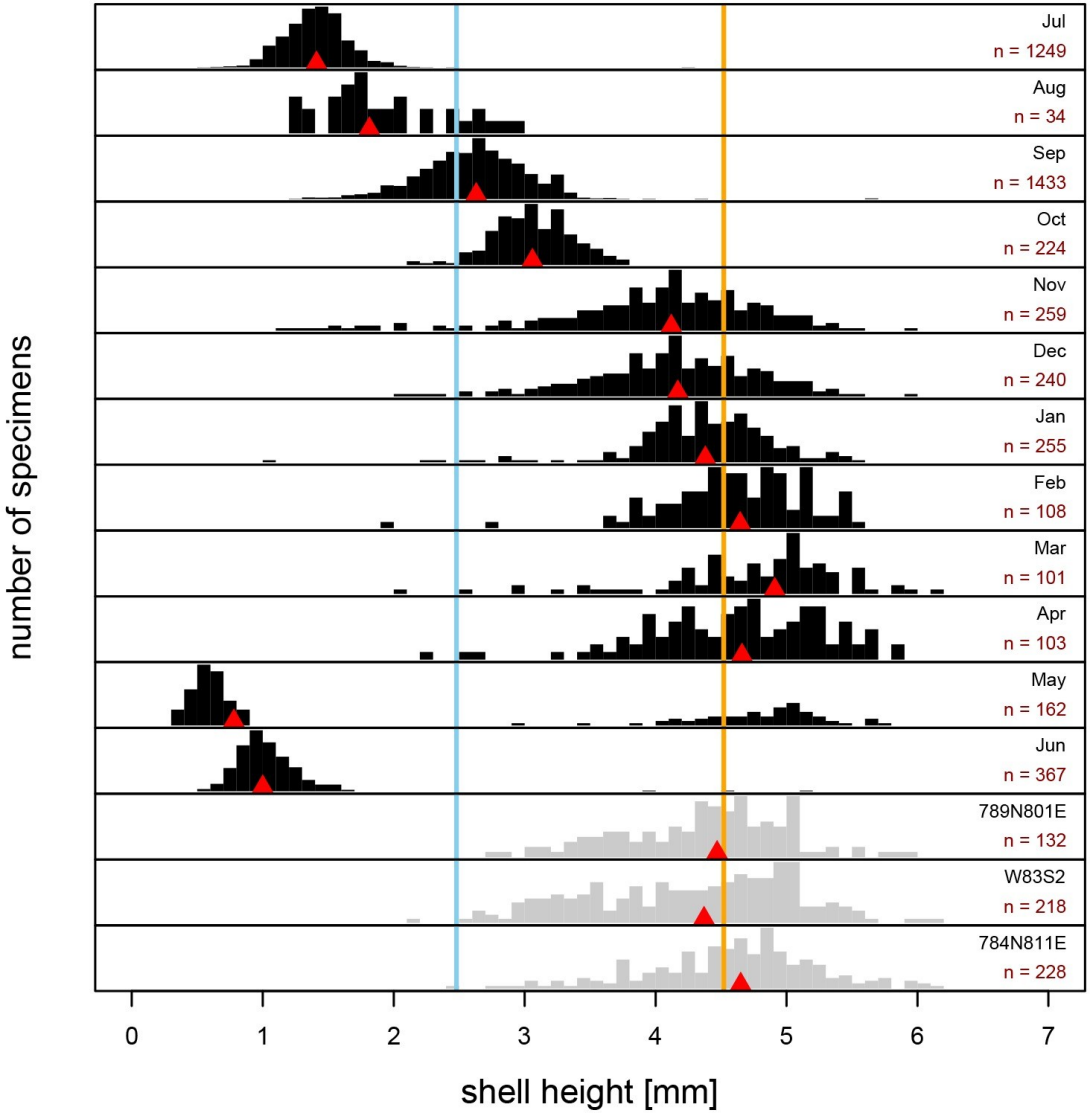
Monthly size-frequency distributions of recent *Boonea impressa*. Size-frequency distributions [R-SFD] of live-collected *Boonea impressa* harvested monthly with data pooled across multiple years (black histograms) compared to size-frequency distributions of the three archaeological samples [A-SFD] (gray histograms). Blue reference line indicates the overall median shell size for all recent data and orange line indicates the overall median of archaeological samples, red triangles indicate median shell size for a given sample. Sample sizes [n] are indicated for each histogram.

**Fig 5:**
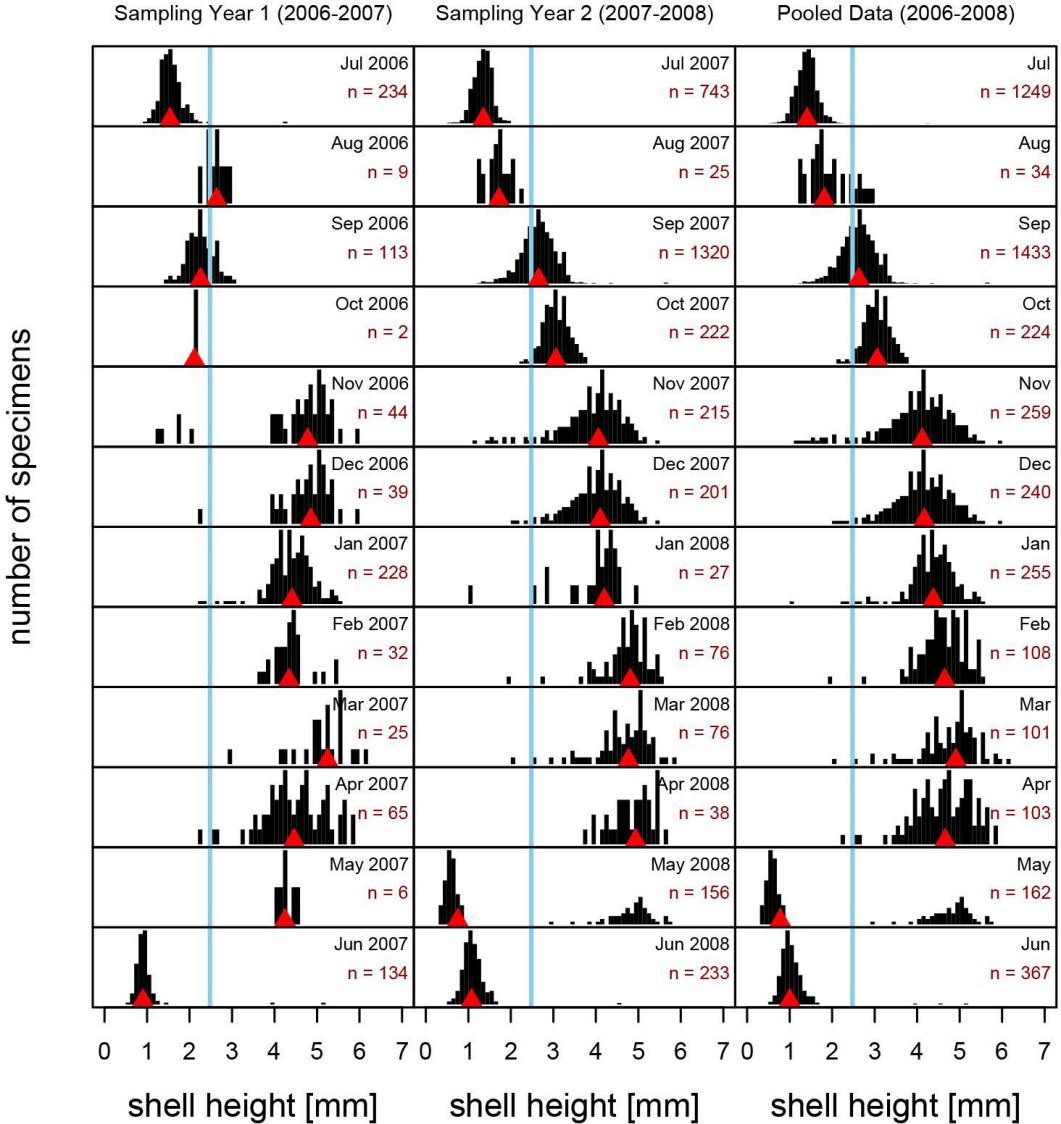
Yearly size-frequency distributions of recent *Boonea impressa*. Size-frequency distributions [R-SFD] of live-collected *Boonea impressa* harvested consecutively for 24 months. Blue reference line indicates the overall median shell size for all data; red triangles indicate median shell size for a given population. Sample sizes [n] are indicated for each R-SFD.

Size-frequency distributions of present-day populations of *Boonea impressa* [R-SFDs], harvested monthly for 24 consecutive months (July 2006 –June 2008), changed successively across seasons (Fig 4). In both years of sampling, a new mode of small specimens appeared in late spring/early summer (June 2007 and May 2008; Fig 5), and the month of May is the only month characterized by a distinctly bimodal distribution, observed in 2008 (Fig 5) and pooled data (Fig 4). The biomodality is not observed for the May 2007 sample, but this may reflect inadequate sampling (*n* = 6). In June, large specimens observed in May samples were nearly completely absent, indicating mortality within ~12 months of spawning in both years of sampling. From June through April, R-SFDs shifted progressively toward larger size classes and remained strongly unimodal (Fig 4). The incremental month-to-month increases in median body size were more notable in summer and early fall, but less pronounced in late fall, winter and spring, as spawning cohorts aged and growth rates slowed (Fig 4). Conversely, the dispersion increased towards fall and winter likely reflecting ecophenotypic variation in growth rates within populations. The increase in dispersion may reflect variability in such factors as the quality of oyster hosts, stunted growth of *B*. *impressa* due to its own parasites, or other processes differentially affecting individual members of the population. Whereas the late fall and winter samples appear visually more similar to each other and medians do not differ notably for consecutive months, the differences in dispersion and dominant age classes makes those distributions subtly distinct. As shown below, these differences are sufficient to produce diagnostic training sets that can be applied to the archaeological samples.

**Fig 6:**
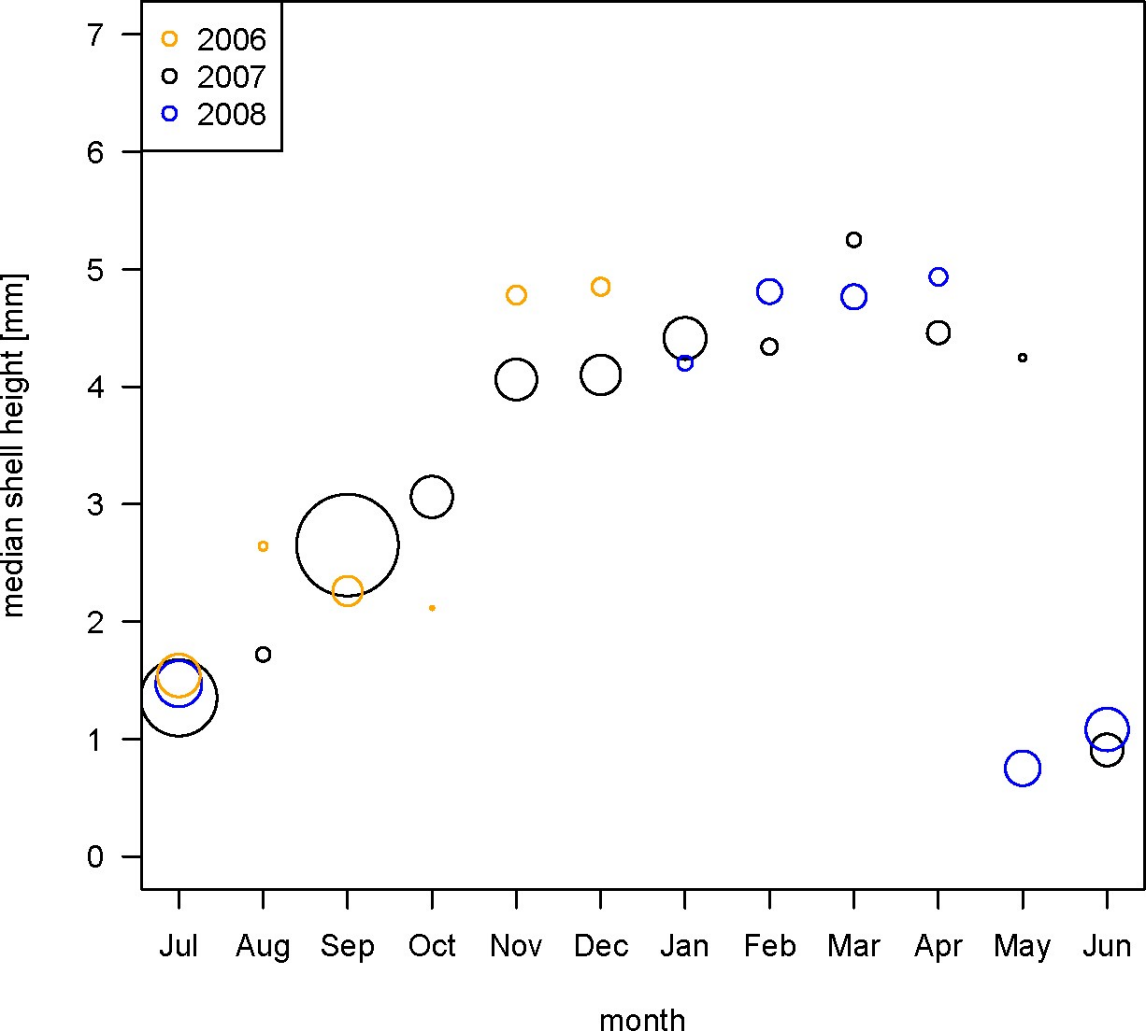
Monthly median body size estimates of *Boonea impressa*. Monthly estimates of median body size of live-collected *Boonea impressa*. Each symbol represents a single month estimate and symbol size is arbitrarily scaled to indicate sample size (number of specimens). Monthly estimates of median size are increasingly consistent when sample sizes of compared monthly samples are reasonably large (see also S1 Fig). Highly discordant estimates involve samples with exceedingly small sample size (May 2007, Aug 2006).

The monthly pattern was highly consistent during the two consecutive years of sampling with median shell height shifting concordantly across months during both sampling seasons, except for rare cases when sample size was exceedingly small for a given month (Figs 5 and 6). Whereas noticeable differences can be observed comparing monthly data across multiple years of sampling (Fig 5), these inconsistencies can be at least partly attributed to sample size. The most dramatic differences in median shell size and between shapes of distributions occur for months in which at least one of the compared samples was represented by a small number of specimens (Fig 6). In fact, a strong inverse rank correlation exists between the magnitude of departure (whether measured by pairwise difference in median size or Kolmogorov-Smirnov D statistic) and the sample size of the smaller of the two compared samples (S1 Fig). That is, only when one of the compared months was severely under-sampled, major discrepancies were observed. In addition to sampling effects, small differences in the timing of spawning events and the minor offsets in the day of sampling in a given month may have induced additional discrepancies between SFDs, especially in months proximal to spawning events.

The results indicate that *B*. *impressa* spawns once a year in late spring and has a maximum lifespan of ~12 months. Due to these two biological characteristics, size-frequency distributions of *B*. *impressa* vary predictably across seasons offering a numerical diagnostic tool for estimating harvest time.

**Fig 7:**
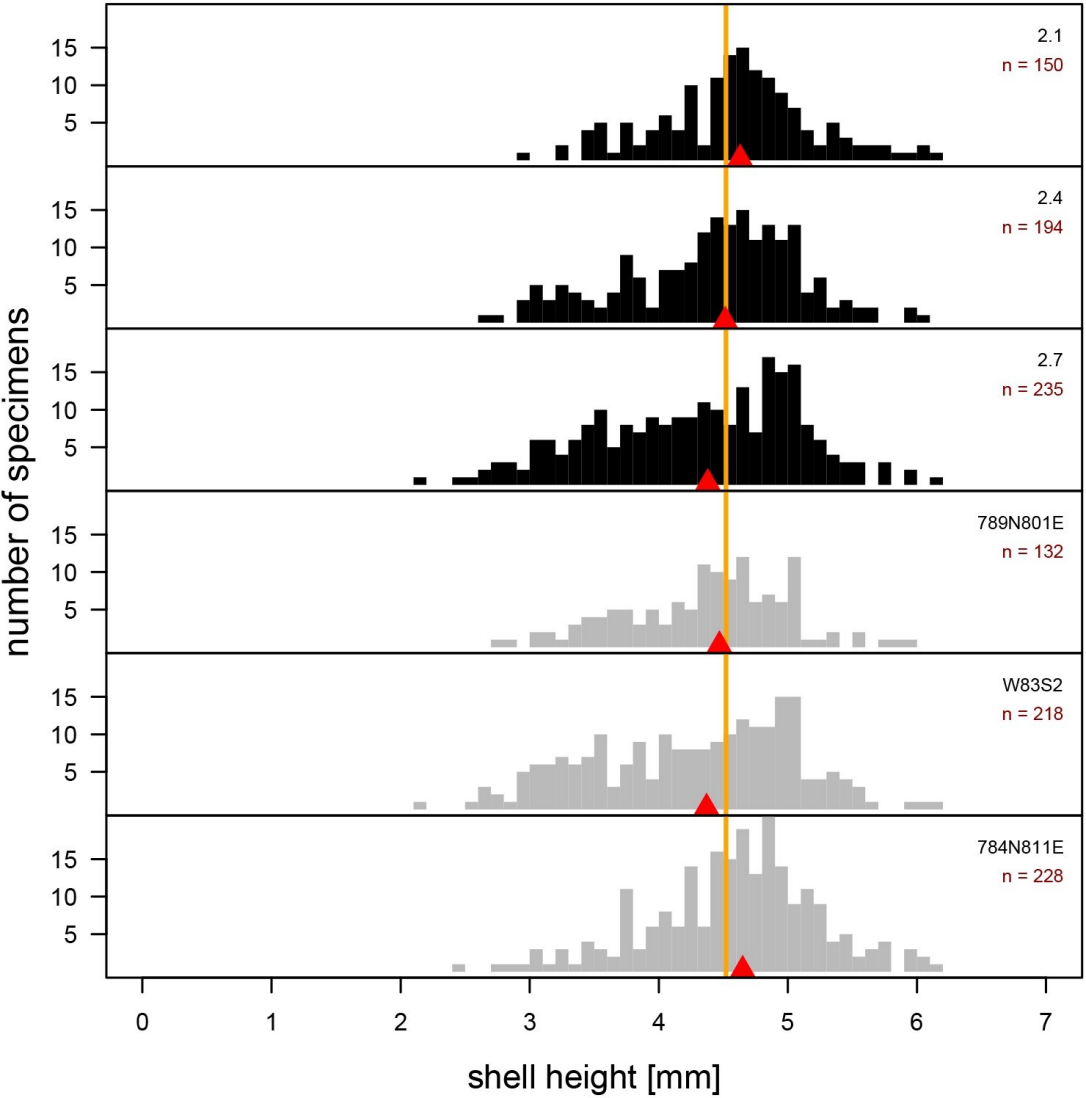
Size-frequency distributions of archaeological specimens of *Boonea impressa*. Size-frequency distribution of archaeological samples derived by two pooling strategies. In the first approach (the three top charts) samples were merged horizontally into three successive stratigraphic slices. In the second approach (the bottom three charts) samples were merged vertically by unit. All samples, whether merged laterally or vertically, yielded highly congruent distributions (see also Table 1).

The three size-frequency distributions A-SFDs derived by pooling archaeological subsamples within each trench (samples 281-W83S2, 784N811E, and 789N801E) are also distinctly unimodal and the maximum specimen size (maximum shell height = 6.20 mm) is comparable to that observed for the present-day populations (maximum shell height = 6.19 mm). The samples derived by horizontal pooling of subsamples (samples 2.1, 2.4, 2.7) are visually comparable to vertical samples (Fig 7). In most pairwise comparisons (15 comparisons total), the three horizontal and three vertical samples cannot be distinguished statistically from one another in terms of the median shell length (66.7% of comparisons) or overall shape of the size frequency distribution (73.3%) (Table 1) and median values of individual samples closely approximate the median value of the entire dataset (Fig 7). Consequently, only vertical samples 281-W83S2, 784N811E, and 789N801E are considered in all subsequent analyses presented below.

**Table 1:** Pairwise comparisons of body size distributions of archaeological samples of *Boonea impressa* derived by either vertical or horizontal aggregation of subsamples.

test	significance (p)	statistic	first sample	second sample	significant	significant with Bonferroni correction
Kolmogorov-Smirnov Test	0.0558	0.145	2.1	2.4	no	no
	0.0010	0.203	2.1	2.7	yes	yes
	0.0331	0.171	2.1	281-W83S2	yes	no
	0.0004	0.219	2.1	784N811E	yes	yes
	0.9767	0.05	2.1	789N801E	no	no
	0.0933	0.12	2.4	2.7	no	no
	0.8131	0.072	2.4	281-W83S2	no	no
	0.0665	0.129	2.4	784N811E	no	no
	0.1423	0.112	2.4	789N801E	no	no
	0.4292	0.095	2.7	281-W83S2	no	no
	0.9967	0.038	2.7	784N811E	no	no
	0.0006	0.188	2.7	789N801E	yes	yes
	0.2191	0.116	281-W83S2	784N811E	no	no
	0.0201	0.166	281-W83S2	789N801E	yes	no
	0.0002	0.203	784N811E	789N801E	yes	yes
Kruskal-Wallis Test	0.0302	4.698	2.1	2.4	yes	no
	0.0011	10.709	2.1	2.7	yes	yes
	0.0057	7.643	2.1	281-W83S2	yes	no
	0.0003	12.893	2.1	784N811E	yes	yes
	0.9701	0.001	2.1	789N801E	no	no
	0.2113	1.562	2.4	2.7	no	no
	0.4634	0.538	2.4	281-W83S2	no	no
	0.1021	2.672	2.4	784N811E	no	no
	0.0174	5.658	2.4	789N801E	yes	no
	0.5897	0.291	2.7	281-W83S2	no	no
	0.6701	0.181	2.7	784N811E	no	no
	0.0004	12.735	2.7	789N801E	yes	yes
	0.3553	0.854	281-W83S2	784N811E	no	no
	0.0031	8.771	281-W83S2	789N801E	yes	yes
	0.0001	15.204	784N811E	789N801E	yes	yes

**Fig 8:**
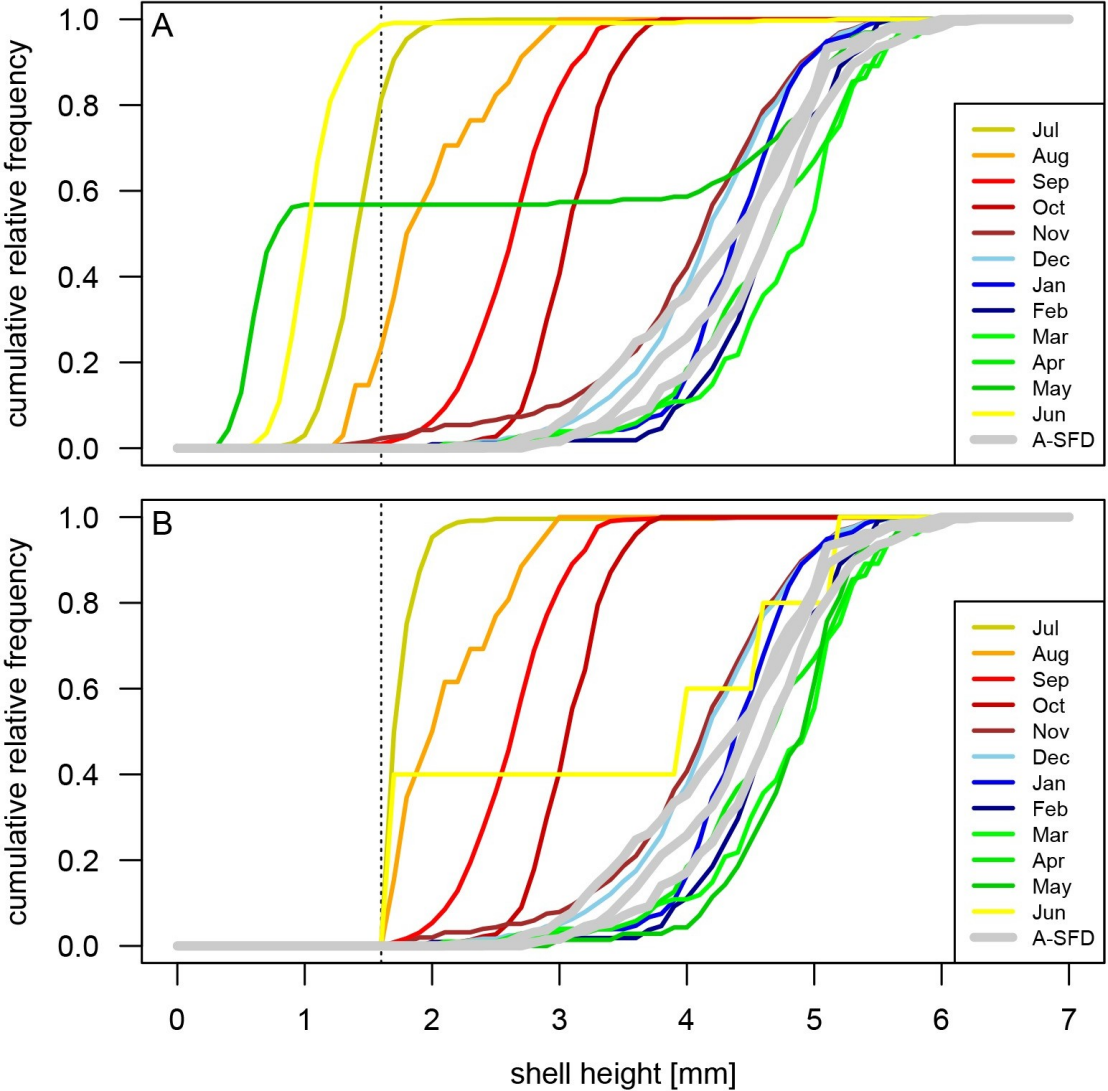
Comparison of recent and archaeological ogives of *Boonea impressa* shell length. Cumulative relative frequency distributions (ogives) of monthly shell size measurements in recent populations of *Boonea impressa* (color-coded by month) compared to the three archaeological samples (thick gray lines) populations. A. Ogives based on all data; B. Ogives restricted to specimens with shell height of 1.6 mm or greater. A dashed vertical line indicates 1.6 mm cutoff value.

The unimodality of the archaeological samples and comparable maximum specimen size are consistent with the assumption that the longevity and growth rate of archaeological populations of *B*. *impressa* were comparable to those observed for the present-day populations. The high abundance of archaeological specimens in size classes that dominate late fall, winter, and early spring samples of recent populations, suggest that archaeological SFDs appear most consistent with the present-day SFDs of mature populations sampled through multiple late fall–early spring months (Figs 4 and 8). The cumulative distribution curves (ogives) estimated from the archaeological samples (Fig 8A) are flatter than those of the recent samples suggestive of a more substantial spread in dominant shell size classes in archaeological samples, intuitively consistent with multiple months of harvesting. However, this visual qualitative assessment of raw data can be further augmented by more rigorous, quantitative modeling of harvest time using samples of recent populations to create single and multi-month reference null models (training sets).

**Fig 9:**
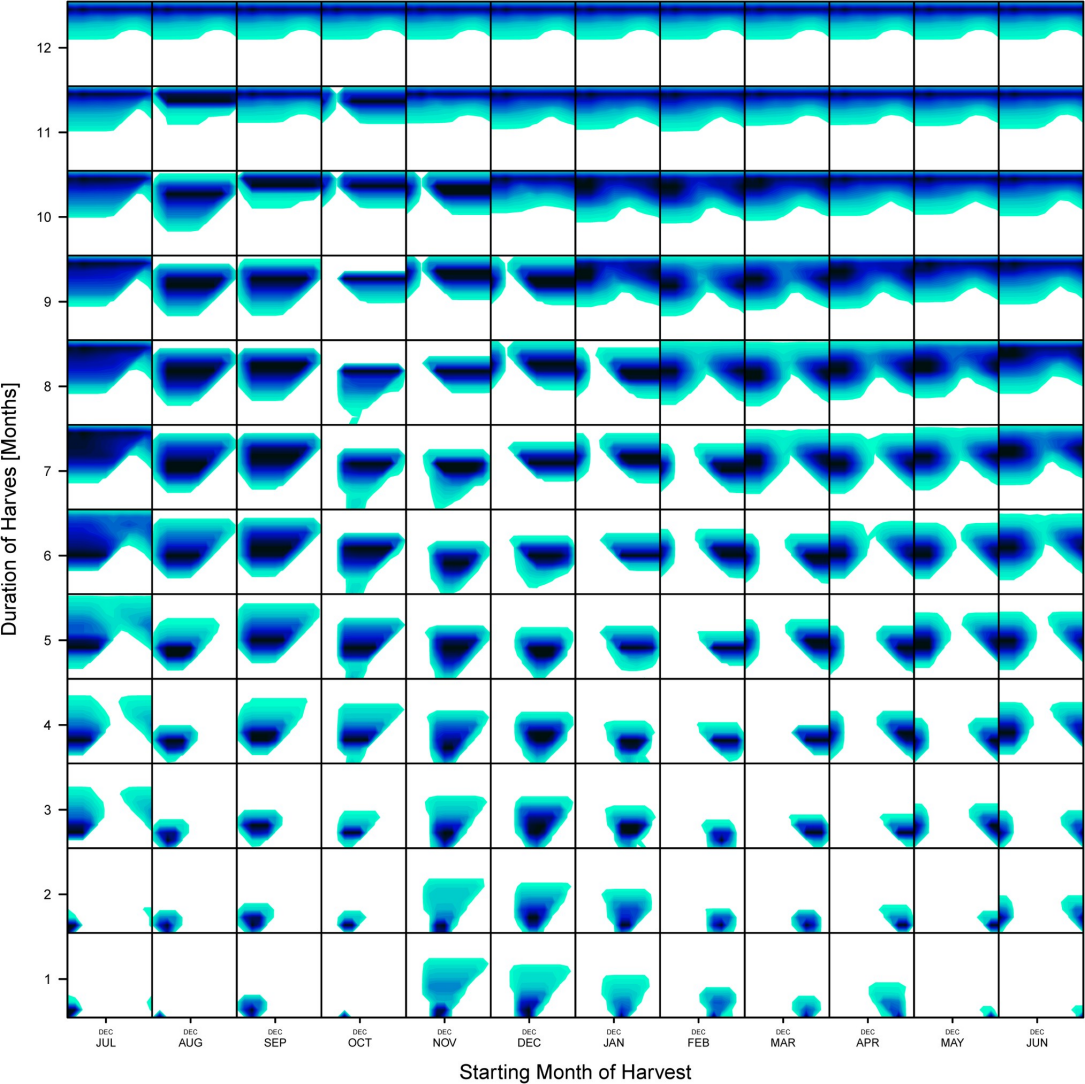
Null models of recent *Boonea impressa* populations. A graphic summary of 133 null models derived from monthly samples of recent populations (Figs 4 and 10, see also S1 Movie). All models exemplified here are based on resampling of a sample of n = 218 specimens (size of sample W83S2). Each box represents a 2D kernel plot documenting distribution of matches (relative frequency of iterations in which the bootstrapped sample of a given month set matched best a given month set) of recent samples of *Boonea impressa*. Those distributions of matches were compared to the distribution of matches derived for the archaeological sample W83S2 and the agreements between the archaeological sample model and null models was evaluated using various measures of similarity (Fig 11).

**Fig 10:**
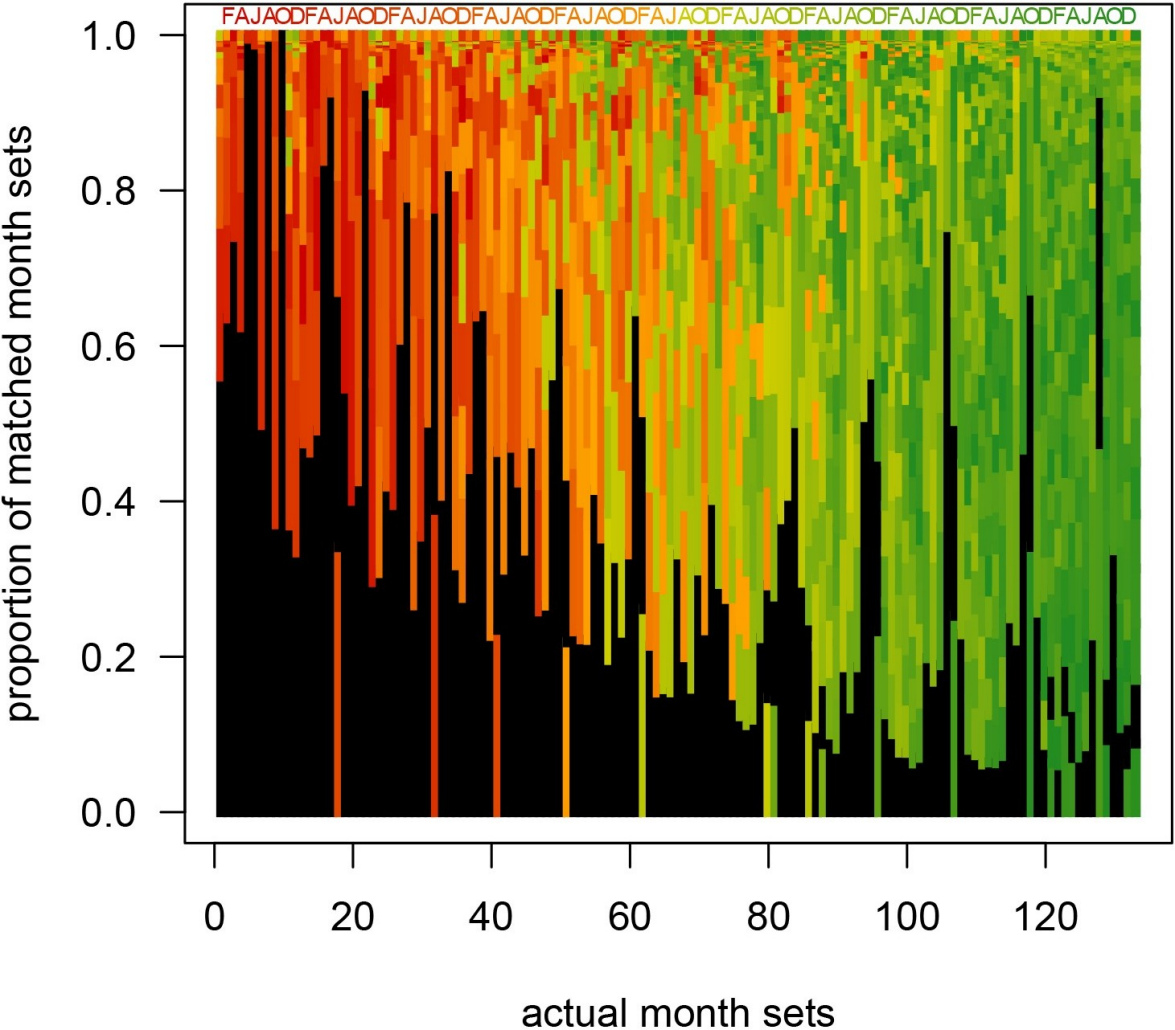
Month set comparisons. Actual month sets (133 month and multi-month combinations) in recent data compared against all possible month sets. The simulation based on the sample size of the archaeological sample W83S2 visually indicates most commonly matched month sets in resampling simulation. Black bars represent the correct match (month set matches itself best). For example, for the second column from the left (1-month harvest in February) ~60% of matches in the simulations match the resampled data correctly as harvested in February. However, other month combinations also occur and consequently the distribution of those specific matches provides a predictive signature for samples collected in 1 month (February harvest). In few cases, (e.g., month set 40 [April-July, 4-month harvest starting in April]), the most frequently matched month set is not itself, but month set 52 (March-July, 5-month harvest starting in March), likely reflecting suppressed effect of outliers. The comparisons of predicted distributions of matches for recent samples with known harvest time (known NULL models) to distributions of matches derived for a given archaeological sample allows to find the best matching null model and the corresponding month set of harvest. Note that for known recent month-sets, short month sets (models 1–36; <4 months of harvest) generally match short month sets (red and orange bars), whereas long month sets (models 97–133, >8 months of harvest) match long month sets (increasingly dark green bars). Mismatches (out of order black bars) become increasingly frequent for longer month sets. Simulation based on 2000 iterations for each of the 133 examined month sets.

**Fig 11:**
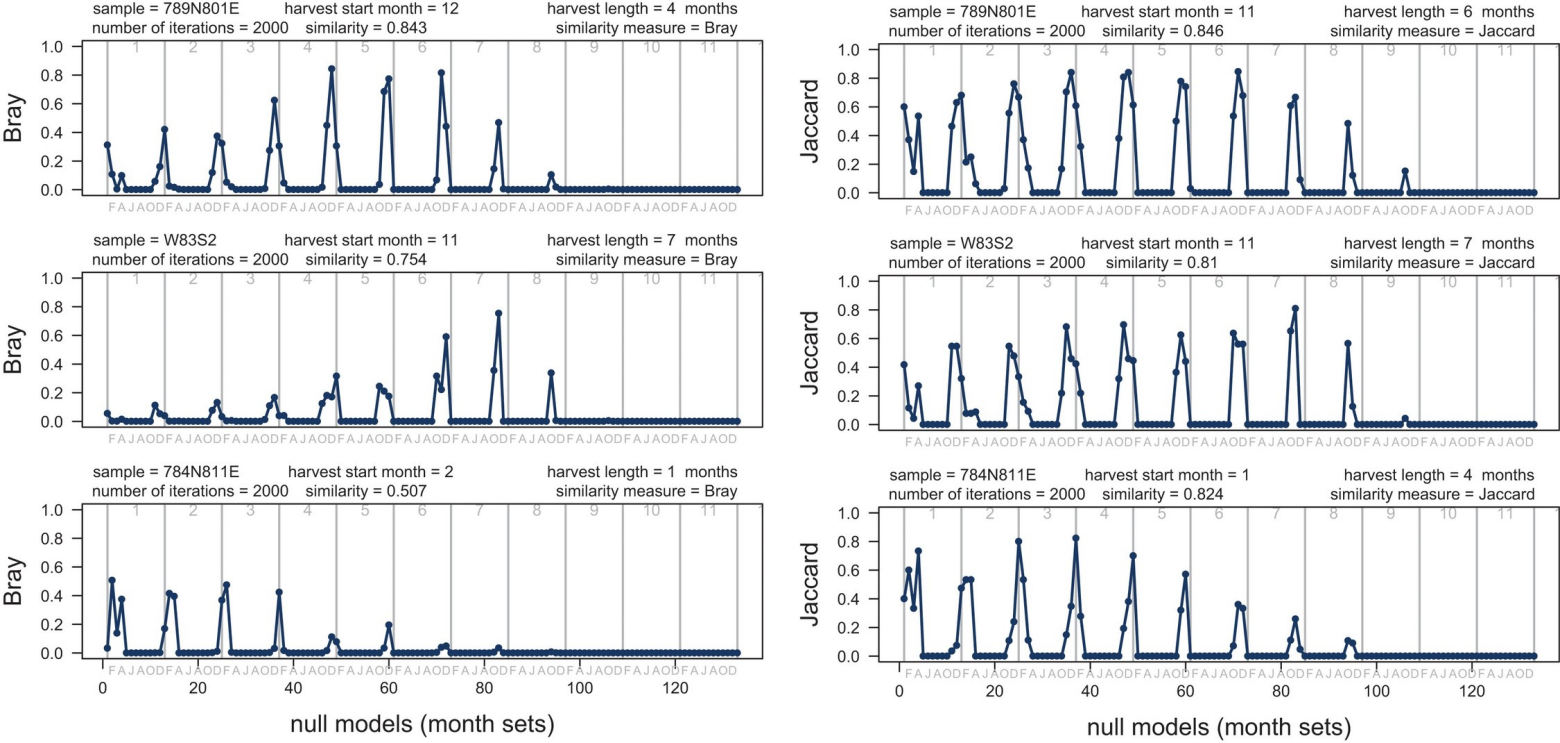
Similarity indices. Comparison of the resampling model for the three archaeological samples to the null models based on 133 recent month sets. Each comparison based on separate 133 null models derived for a sample size of a given archaeological sample. Similarity between the null models (distribution of matches) predicted for recent month sets (Fig 10) and the model matches for archaeological samples is measured using a presence-absence similarity measure (Jaccard, left) and an abundance-based similarity measure (Bray-Curtis, right) See also (Figs 5 and 13). Note that peaks (high similarity) mostly cluster for harvest starting in late fall (November-December) and occasionally early winter (January). Highest peaks (best model matches) suggest multi-month harvest. Gray numbers (tops of charts) represent month sets of given duration and gray letters (chart bottoms) represent start months (only even months shown): F-February, A-April, J-June, A-August, O-October, D-December.

The series of 133 reference month sets SFDs ranged from SFDs representing a single-month harvest to SFDs representing 12 months of harvest. The 133 models (Fig 9, see also S1 Movie 1) developed by matching each replicate reference month set to all 133 reference month sets represent null predictions (training sets) when the harvest time is known (see S1 Appendix for details). For each archaeological sample, the separate set of 133 models was developed for the sample size *n* of the evaluated archaeological sample (only one of the three sets of models is illustrated here; Fig 9). For most month sets, the most frequent match is not surprisingly that month set itself. However, substantial numbers of matches to other month sets also occur for all month set models (Fig 10). Consequently, each of the 133 null models provides a unique quantitative signature of month set matches (Figs 9 and 11) that can be than used as a comparative training set for assessing each archaeological sample.

**Fig 12:**
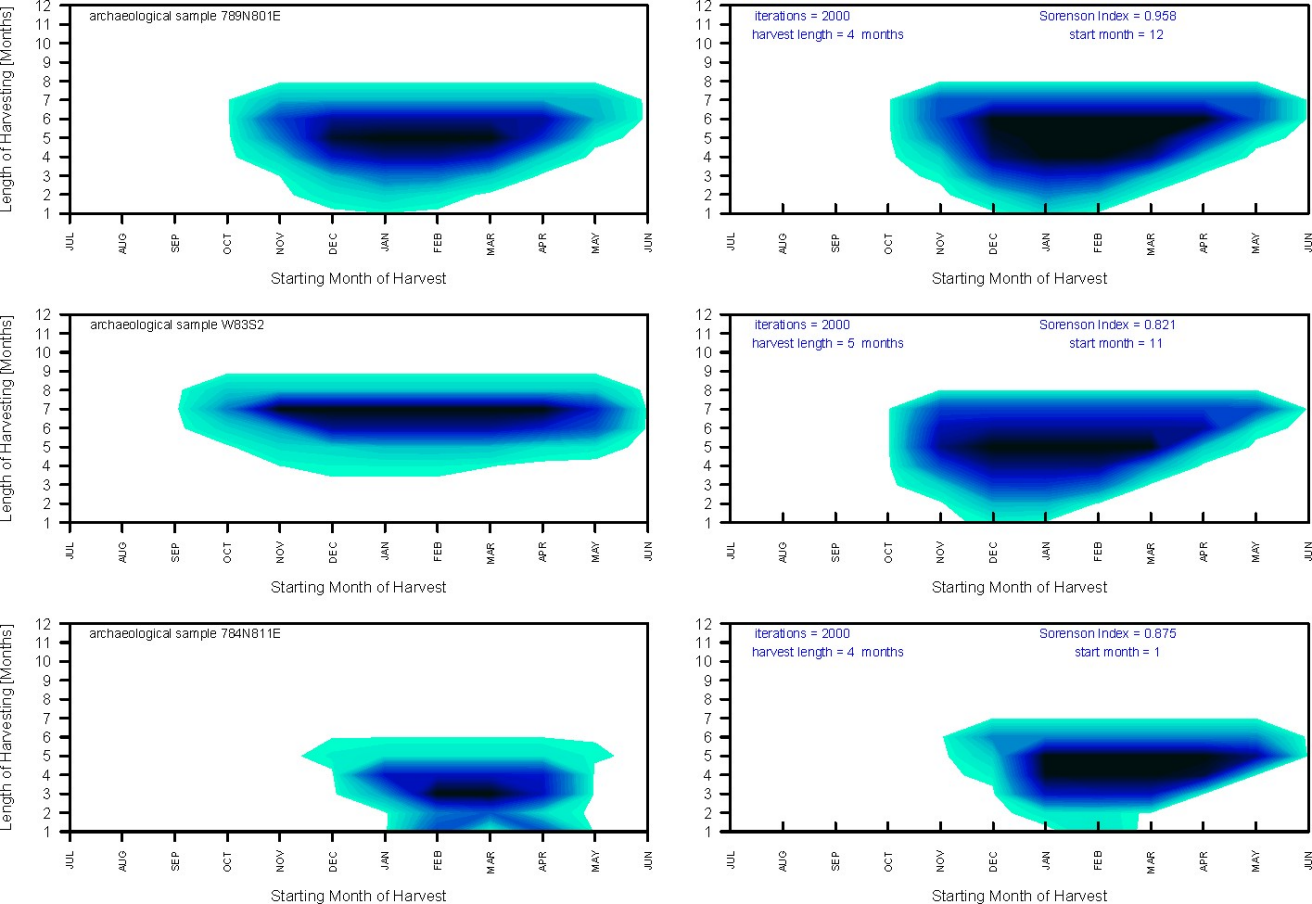
2D-kernel density distribution. The 2D-kernel density distribution of month set matches for the archaeological sample (left panel) and the best-matched null model (right) selected based on Sorenson similarity. All other closely matching null models also suggest (Fig 13) that sampling started in late fall (November or December).

**Fig 13:**
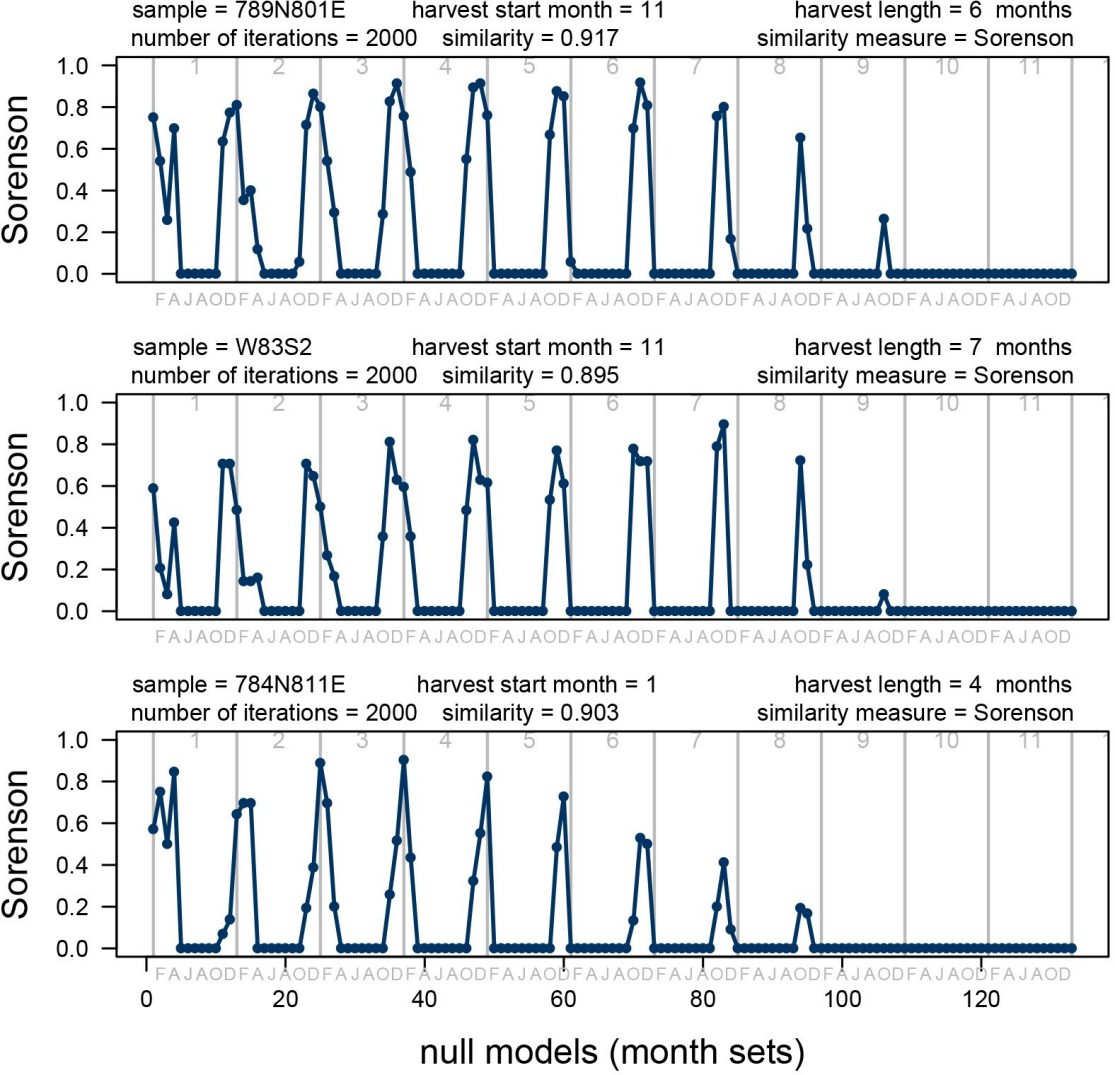
Sorensen similarity index. Comparison of the resampling model for the three archaeological samples to the null models based on 133 recent month sets. Each comparison based on separate 133 null models derived for a sample size of a given archaeological sample. Similarity between the null models (distribution of matches) predicted for recent month sets (Fig 10) and the model matches for archaeological samples is measured using presence-absence similarity measure (Sorensen). Similar results are obtained for Jaccard (Presence-Absence) and Bray-Curtis (abundance) similarity measures (Fig 11). Note that peaks (high similarity) mostly cluster for harvest starting in late fall (November-December) and occasionally early winter (January). Highest peaks (best model matches) suggest multi-month harvest. Gray numbers (tops of charts) represent month sets of given duration and gray letters (chart bottoms) represent start months (only even months shown): F-February, A-April, J-June, A-August, O-October, D-December.

**Fig 14:**
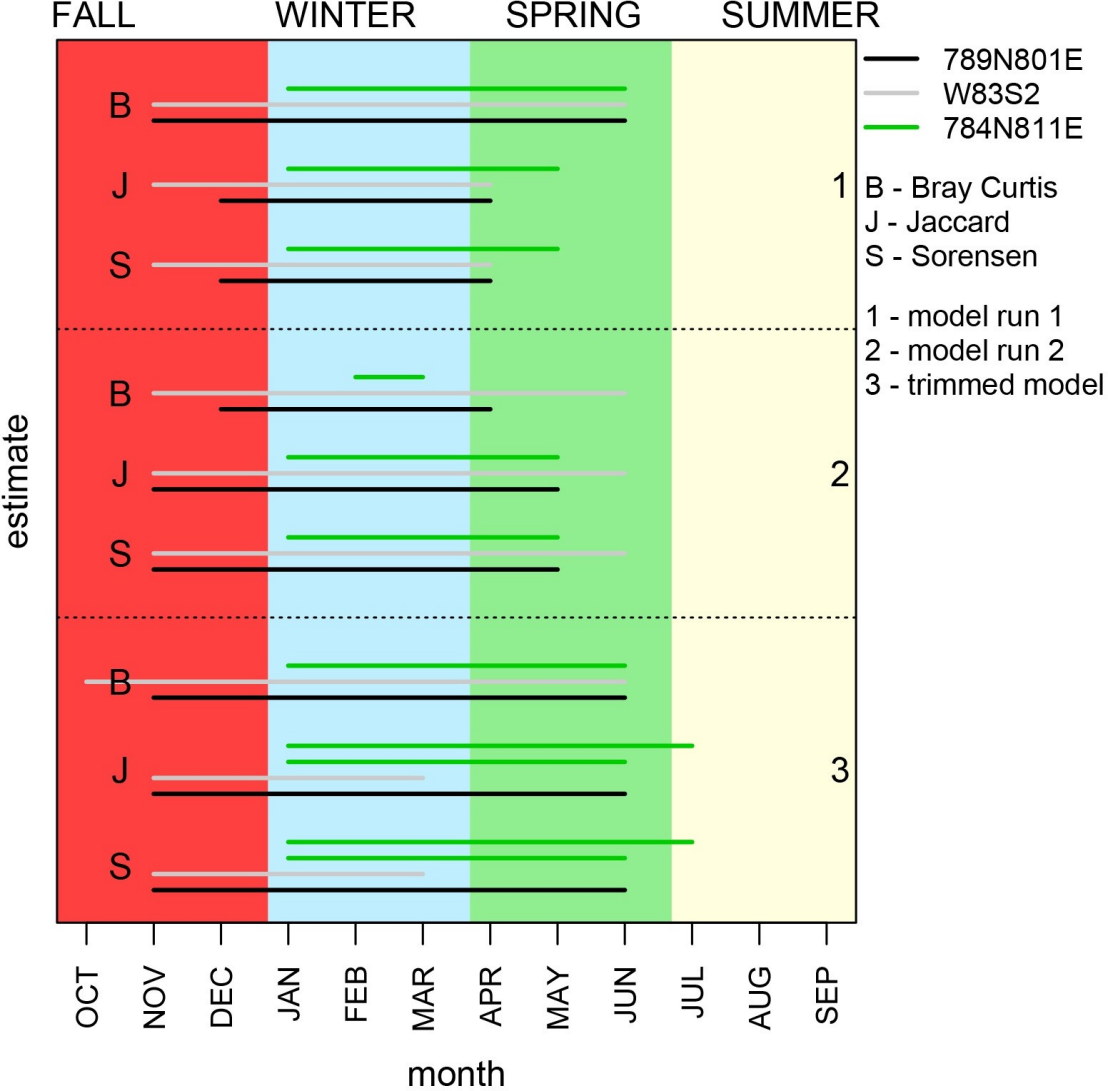
Comparison of resampling models for full data set versus trimmed data. Comparison of repeated runs of resampling models for the three archaeological samples. The bars represent the most likely harvest timespan suggested in a given simulation. Colors of bars differentiate estimates for three different samples. The outcomes of each simulation was evaluated using three different measures of similarity (“B” = Bray-Curtis, “J”= Jaccard, and “S” = Sorensen). Estimate sets “1” and “2” represent repeated runs of simulations based on all recent specimens, including those smaller than 1.6 mm. The estimate set “3” represents simulations based on the recent dataset restricted to specimens with minimum length of 1.6 mm. Multiple bars for the same sample (“3”, “J”, green and “3”, “S”, green) represent two estimates of harvest time that both yielded highest similarity coefficients.

The archaeological sample 789N801E (n = 132) best matched the null model for a harvest period of four months starting in December and ending in March (Fig 12). The sample 281-W83S2 (n = 218) best matched the null model for a collection period of five months starting in November and ending in March (Fig 12). The sample from 784N811E (n = 228) best matched the null model for a harvest duration of four months starting in January and ending in April (Fig 12). Overall, null model matching indicates that all three archaeological samples fit best models suggesting 4 to 8 months of harvesting starting in the late fall or early winter. Other null models with high similarity (similarity index > 0.5) also consistently suggest onset of harvest in late fall/early winter and multi-month harvesting season (Fig 13). The null models that include summer months tend to yield similarity indices nearing 0, indicating that the archaeological samples are unlikely to include oysters collected in summer (Fig 12). The results can be replicated consistently for multiple similarity measures (Figs 11 and 13). Also, the repeated set of simulations yielded highly congruent outcomes (Fig 14).

When specimens measuring <1.6 mm were removed from the recent dataset–to determine whether season of harvest estimates are affected by disparities between sampling methods used to process archaeological and live population samples–the results remained consistent (Fig 8B and 14). Again, for all three samples the suggested harvest time started in late fall and winter and lasted for multiple months (Fig 14).

All above results are limited to continuous multi-month models. Whereas exploring all possible discontinuous permutations is computationally prohibitive, it is instructive to consider discontinuous variants of best continuous models. This supplementary analysis (S2 Fig) suggests that the best continuous models outperform all its discontinuous variants for two out of three samples: archaeological samples 789N801E and W83S2. However, for archaeological sample 784N811E a discontinuous model (February and April harvest) outperforms the best continuous model. In addition, for sample 789N801E the best discontinuous model (December-February-March) performs nearly as well as the best continuous model (December-March). This outcome should serve as a cautionary note suggesting that whereas multi-month harvesting is consistently supported in nearly all analyses presented here, it is less certain whether oyster-gathering activities occurred continuously throughout the entire multi-month interval of harvest and the actual duration of harvest is unknown.

While the results are consistent with multi-month harvest, this may be also due to time-averaging of short-term harvest events. For example, the apparent multi-monthly harvest could easily be produced if short-term harvest events underwent time-averaging over multiple decades or centuries. Slight differences in harvest time, spawning time, growth rate (induced by year-to-year environmental fluctuations or other causes) could increase dispersion of the time-averaged SFD and make samples more consistent with multi-monthly demographic training sets. Thus, the results suggest that harvesting was strongly seasonal, but the time-averaged data (and resulting model outcomes) may overestimate the harvest duration. Nevertheless, data consistently suggest that the harvest was not likely to start earlier than November or continue past May. The modeling exercise also demonstrates that archaeological samples are highly incongruent with any substantial harvest activities during summer and early fall.

## Discussion

### *Boonea impressa* as a seasonal indicator

The use of *Boonea impressa* as a proxy for archaeological season of occupation has been criticized [18] because the approach invokes multiple assumptions about the odostome life cycle and behavior. Specifically, the method assumes that the odostome life cycle is spatially and temporally invariant (implicitly positing that the spawning time does not vary notably from year to year and from region to region), multiple spawning events do not occur annually, growth patterns within populations are invariant, and endoparasitic feeding behavior is obligatory for both juveniles and adults [13,18]. Our quantitative surveys of recent populations indicate (Figs 4, 5, 6 and 8A) that the contemporary odostome populations from St. Catherines Island meet the key assumptions: local odostome populations live for only one year, show a mostly consistent growth pattern (when sampled adequately) over the continuous two-year collection period, and spawning occurs only once during the year. The use of *B*. *impressa* is further justified here because recent samples came from oyster reefs located in close proximity to the shell ring (spatial sympatry). Moreover, the Late Archaic time interval represented by the shell ring corresponds to an earlier phase of the current interglacial when the climate regime is expected to have been comparable to current conditions. In addition, the largest archaeological specimens closely match the largest specimens from present-day populations, and archaeological size-frequency distributions demonstrate excellent matching with some of the predictive models (excellent model matching would be unlikely if recent and archaeological samples came from populations that grew at notably different rates or varied in spawning and mortality patterns).

### Implications of seasonal oyster harvest at St. Catherines Shell Ring

Previous research on St. Catherines Island has focused on seasonal settlement and mobility patterns from the Late Archaic to the historic period [30]. However, the timing of oyster harvest has not been investigated rigorously despite the fact that oysters are the most frequently identified invertebrate taxon from St. Catherines Shell Ring [19]. Oysters likely played a significant role in the subsistence and social economy of the communities living at, or regularly visiting, the ring (see [30]). Our results indicate that the oysters were harvested primarily during late fall, winter and spring suggesting that ring formation occurred primarily during the cool-weather months. These interpretations are congruent with sclerochronology of hard clams (*Mercenaria* spp.) from St. Catherines Shell Ring, which points to predominant winter/spring harvest [31]. Similarly, botanical remains indicate most intensive use during the fall [32]. Our results suggest that harvesting concentrated in non-summer months. However, the continuity and exact duration of these seasonal harvest events is more difficult to assess due to time-averaging. Also, the results do not necessarily imply that the ring was not used during other times of the year. The presence of vertebrate and archaeobotanical remains of organisms that were likely available only during the summer and fall indicate that smaller populations may have remained at the ring during this time [24,32]. However, it is likely that the primary ring building activities occurred during winter and spring. The fact that this seasonal pattern of use is observed for all three vertically aggregated archaeological samples that span the entire temporal record of the shell ring suggests that the harvest patterns were already established at the inception of the ring and maintained over multiple centuries. Since the samples are time-averaged, changes in harvesting patterns that may have occurred at shorter time scales are difficult to detect.

It is noteworthy here that because of limited sample sizes, the samples had to be pooled (either vertically or laterally) limiting the interpretative power of these analyses. It is reassuring that whether samples were pooled vertically (substantial analytical time-averaging) or horizontally (in which spatial and analytical time-averaging both occur), the resulting pooled samples produced consistent outcomes. Analyses based on more extensive sampling of single horizons could further improve the interpretative power of the proposal approach, although it should be noted that even if the data were constrained to one well controlled horizon, the within-horizon time-averaging would still likely be substantial.

The seasonal pattern of oyster harvest, augmented by other lines of evidence reported in previous studies [33] is congruent with the hypothesis that human activities at St. Catherines Shell Ring fluctuated seasonally. Local and regional communities may have converged for seasonal gatherings during the spring, winter and fall, while during summer months only a small portion of the population remained at the rings [32]. Under this scenario, the mass deposition of oysters during fall-spring intervals reflects a continuous or discontinuous period of use and deposition throughout the cool weather months, repeated for centuries. Recent studies in the seasonal collection of various vertebrate, invertebrate and plant species from St. Catherines Shell Ring also indicate multiple seasons of use with varying intensity of occupation throughout the year [33]. We stress here that, on its own, the seasonal oyster harvest is not a conclusive line evidence for a seasonal occupation of shell rings, as it may also reflect seasonal shifts in preferred food sources (see below). However, the seasonal oyster gathering practices are congruent with previously reported data that point to diminished human presence during summer months at this [32] and other [34] shell rings.

Due to the limited number of sites with oyster seasonality data, few comparisons can be made with other shell ring sites and Archaic period midden sites. Nevertheless, a similar pattern for oyster harvest is evident at the Sapelo Shell Ring complex (Sapelo Island, Georgia), where stable isotope analysis of oyster shells suggests a seasonal harvesting pattern that was primarily restricted to winter months [34]. However, oyster isotope data taken as a whole indicate year-round harvest site-wide [34].

### Seasonal consumption of oysters

The pattern of oyster harvest exhibited at St. Catherines Shell Ring supports the hypothesis that oysters were harvested seasonally, during the cooler months of late fall, winter and spring and this pattern persisted through time. That Archaic people did not consume oysters during summer months aligns with the old adage that oysters should only be eaten in the months with the letter “r”, but avoided during all other months [35]. Today, during the warm summer months, oysters harbor increased levels of pathogenic bacteria that may result in serious illness or, in some cases, death [36]. In addition, oysters in the southeast spawn from May through October [37] during which time stored glycogen is used to produce gametes. Once released, oysters become watery in texture and unpalatable [38] until late December or early January when meat yield increases [30]. Archaic peoples could have avoided harvesting and eating oysters during the warm, spawning months of summer because they were aware of potential health risks and shared our dislike for watery, poor quality meat. It has also been suggested the people of St. Catherines Island may have practiced some form of mariculture [39], so avoiding oysters during the summer months may have served to manage and maintain the viability of oyster populations. Allowing oysters to spawn during the summer without significant harvesting pressure would have allowed beds to recover after big harvests in the winter and spring, ensuring long-term sustainability of the resource. Finally, it may be possible that oyster harvest ceased during summer months as people at the ring focused on other seasonal food sources.

## Conclusion

The modeling approach developed here provides numerical support for the hypothesis that shellfish were deposited seasonally, and this practice may have been the primary means by which Archaic period people formed St. Catherines Shell Ring. When appropriately evaluated and sampled, *Boonea impressa* may serve as a reliable proxy for the determination of oyster harvest seasons. When determining seasonal site use or occupation, the proxy can be particularly useful when correlated with other critical lines of evidence such as stable isotopic data, use patterns for other animal and plant remains, and stratigraphy.

Archaic shell-ring dwellers at St. Catherines Island, harvested oysters primarily during the late fall, winter and/or early spring. This gathering pattern matches seasonal harvesting postulated for other mollusks (clams) at this and other Archaic shell rings where similar studies have been conducted. Oyster consumption was avoided during summer months, possibly reflecting poor palatability, pathogen avoidance, or perhaps one of the earliest records of sustainable harvesting. Oysters, as ecological keystone species, are important indicators of estuarine health but their numbers are currently on the decline worldwide [40–42]. Archaeological assessments of intensity, seasonality, and sustainability of oyster harvesting in the past provide us with a unique perspective on the long-term history of human-oyster interactions. These type of studies are increasingly used toward developing a more robust understanding of historical ecology of estuarine and coastal systems for various regions of the southeastern USA, including the Chesapeake Bay [43] and the gulf coast of Florida [44,45]. How past human populations may have mitigated problems associated with overharvesting or resource depletion has important implications for contemporary issues regarding the sustainability of estuarine environments in the face of environmental degradation and human harvesting pressures.

## Supporting information

S1 Appendix Supporting information. Statistical modeling approach, methodology, zooarchaeological data, and *Boonea impressa* specimen repository information.
(PDF)

S1 Movie 133 null models. Animation showing a sequence of 133 null models depicted in Fig 9.
(GIF)

S1 Fig Similarity indices for discontinuous models. Comparison of the performance of best continuous models to their discontinuous variants. Comparisons performed separately for each of the three archaeological samples. X-axis labels indicate the discontinuous month combinations. Red point marks the best continuous model for each sample and the dashed line represent the similarity index value for that best continuous model. All models evaluated using Bray-Curtis similarity index.
(TIF)

S2 Fig Comparison of shell length and size frequency distributions. Difference between monthly size-frequency distributions of live-collected specimens of *Boonea impressa* plotted as a function of sample size (n) of the smaller of the two compared monthly samples. Each data point represents a pairwise comparison for the same month from different years (e.g., March 2007 vs. March 2008). A. Pairwise differences in median shell length between the two compared years for a given month. B. Kolmogorov-Smirnov D statistic measuring overall difference in the shape of the two compared size-frequency distributions.
(TIF)
