# Supplementary material for: Seasonal oyster harvesting recorded in a Late Archaic period shell ring
Source: PLoS One. 2019 Nov 20;14(11):e0224666. doi: 10.1371/journal.pone.0224666 (PMC6867601; doi:10.1371/journal.pone.0224666)
Supplement: S1 Appendix — Statistical modeling approach, methodology, zooarchaeological data, and Boonea impressa specimen repository information. (PDF) [file pone.0224666.s001.pdf]

Supporting Information

Appendix 1

Statistical Modelling Approach

An approach proposed here provides a quantitative assessment of the most likely harvest time of oysters in Archaic shell rings. Specifically, we compare size-frequency distributions of parasitic snails *Boonea impressa* derived from archaeological samples (unknown harvest time) to recent size-frequency distributions derived by monthly sampling of living populations (known harvest times). In this approach, that represents a simple machine-learning strategy, a model derived for an archaeological sample is compared against all possible null models derived from live-collected samples. A total of 133 unique consecutive month sets can be constructed for monthly datasets (see below). Consequently, 133 null models (or training sets) can be developed as reference standards to which the model of a given archaeological sample is compared. We do not consider here non-consecutive month combinations because this approach is computationally prohibitive and, more importantly, because the consecutive-month models performed well in the final analyses. Pairwise similarity measures are then used to choose the null model that best matches the model signature of the archaeological sample, an approach somewhat analogous to the Maximum Likelihood approach. The best matched null model represents the most likely harvest time for a given archaeological sample.

The following empirical values and datasets are used here:

N_K – Sample size (number of specimens) of an archaeological sample K (3 samples total)

R-SFD – a size-frequency distribution of live-collected specimens of *Boonea impressa* (shell height measurements). R-SFDs include not only single-month R-SFDs (Fig. 5), but also all possible consecutive month set combinations derived by pooling 12 R-SFD samples across one or more consecutive months. There are 144 consecutive month sets from January to December:

1 month of harvest
- Month Set 1: Jan
- Month Set 2: Feb
- (. . .)
- Month Set 12: Dec

2 month of harvest
- Month Set 13: Jan + Feb
- Month Set 14: Feb + Mar
- (. . .)
- Month Set 24: Dec + Jan

3 months of harvest
- Month Set 25: Jan + Feb + Mar
- Month Set 26: Feb + Mar + Apr
- (. . .)
- Month Set 36: Dec + Jan + Feb

4 months of harvest

Month Set 37: Jan + Feb + Mar + Apr
Month Set 38: Feb + Mar + Apr + May
(. . .)

(…)

12 months of harvest
Month Set 133: Jan + Feb + Mar + Apr + May + Jun + Jul + Aug + Sep + Oct + Nov + Dec
(. . .)
Month Set 144: Dec + Jan + Feb + Mar + Apr + May + Jun + Jul + Aug + Sep + Oct + Nov

Because month Sets 133 – 144 are identical (12 R-SFDs pooled into a single dataset), only 133 R-SFDs (month sets) are considered in the modeling protocol.

R-SFD* – a bootstrap sample (N_K) of a given size-frequency distribution (R-SFD) of recent specimens of *Boonea impressa* (shell height measurements). For each R-SFD, R-SFD* with sample size N_K is generated by resampling with replacement. Note that for each archaeological sample, separate R-SFD*s need to be generated to reflect the sample size N_K of that archaeological sample.

A-SFD - a size-frequency distribution of a given archaeological sample of *Boonea impressa* (shell height measurements) (Fig. 4)

A-SFD* - a bootstrap sample of size-frequency distributions of archaeological samples of *Boonea impressa* (shell height measurements). For a given A-SFD with sample size N_K, A-SFD* with the same sample size N_K is generated by resampling with replacement.

Training Sets (Null Models): For each archaeological sample, a set of 133 null models (each possible consecutive month combinations) was generated using the following protocol:

1. For a given month set (e.g., January-February R-SFD [R-SFD_{13}]), observed shell height values was resampled with replacement (bootstrapping) using the sample size N_K of the archaeological sample under evaluation. Because three archaeological samples have been analyzed, three separate training sets of models (for three unique N_K values) were constructed.

2. The resulting R-SFD* sample (e.g., R-SFD_{13}*) was compared successively against each of the observed 133 R-SFDs. A Kolmogorov-Smirnov statistic D was used to measure similarity between the evaluate R-SFD* and all observed R-SFDs. The R-SFD comparison that yielded the smallest D value was recorded as the best match. If multiple minima were observed, all R-SFDs that produced minimum D-value were recorded. For example, if a bootstrap sample R-SFD_{13}* returned minima with R-SFD_1 and R-SFD_{25}, both matches were recorded.

3. For each month set (e.g., R-SFD_{13}), the 2000 R-SFD* replicates were generated to produce a list of best matched R-SFDs. Because multiple minima were recorded for most R-SFDs, the list of best matches often exceeded 2000. For each R-SFD, a unique model of 2000 or more matches was produced. For example, for archaeological sample W83S2, the month set R-SFD_8 (1-month harvest in August) produced 2000 R-SFD_8 matches (a perfect match). However, for most R-SFDs, best matches represented a distribution of multiple R-SFD matches (Fig. 10). The distribution of matches (e.g., 1077 R-SFD_{13}, 600 R-SFD_{25}, 369 R-SFD_1, 35 R-SFD_{37}, 12 R-SFD_{49}) represents a training set: an expected distribution of matches for a sample with known harvest time. These training sets or null models are predicted distributions of matches. They can be visualized as stacked frequency charts (Fig. 10) or 2D-kernel density plots (Fig. 12). For each archaeological sample, a set of 133 training sets (or null models) were derived by resampling(

Fig. 9). It is noteworthy that while the known R-SFDs are usually most frequently matched correctly, in some cases simulated R-SFD*s matched wrong R-SFDs more often (Fig. 10). This is most likely due to the fact that the role of outliers is reduced in simulations for pooled data (thus, January R-SFD may match January-February data better than January data because outliers are resampled less frequently with increased sample size). The final interpretations are based on matching archaeological models against null models (and not original R-SFDs), and thus these spurious sampling biases are avoided.

Evaluation of Archaeological Samples

4. For each archaeological sample the same protocol (steps 1-3 above) was employed to generate null models/training sets (2000 iterations). In this case 2000 A-SFD* samples were compared against all original 133 R-SFDs. This procedure generated one distribution of matches for each archaeological sample, which can be then compared against all 133 training sets for known null models.

5. Each of the 133 null models generated for a sample size of a given archaeological sample, was compared to the model generated for that archaeological sample. The pairwise similarity between each null model and the archaeological sample model was measured using Bray-Curtis (abundance similarity measure), Jaccard Similarity (presence-absence similarity measure), and Sorensen Similarity (presence-absence similarity measure). In the case of the Bray-Curtis measure, similarity of 1 is attained where distributions of best matched R-SFD is identical in terms of frequency of replicate samples. In the case of Jaccard and Sorensen indices, similarity of 1 is attained when the same set of R-SFD is found for the two compared models regardless of relative frequency of matches. The three indices yielded qualitatively comparable results for all three archaeological samples (Figs 11, 13, and 14).

Methodological Notes

The step 5 is somewhat analogous to Maximum Likelihood in that it evaluates multitude of null models against the observed data. However, in the approach employed here the decision is based on a maximized model similarity rather than a formally maximized likelihood.

Because all null model evaluations are based on the same number of parameters and the same sample size (given by the evaluated archaeological sample) parameter/sample size weighting (analogous to AIC or BIC) is not relevant.

The choice of 2000 iterations reflects a computer intensive nature of the approach. For a single archaeological sample, 2000 iterations translate into 133*133*2000 (3537800) iterative computations of D values. Repeated 2000 iteration runs yielded consistent results indicating that increasing the number of replicate samples was not necessary.

The similarity values are interpreted here as measuring the strength of quantitative support for a given model. Consequently, all models with high similarity measures need to be considered as tenable. We thus acknowledge that multiple possible harvest times can be postulated for each of the analyzed archaeological samples. However, all models with high similarity values consistently suggest that (1) samples represent multiple months of harvest; (2) harvest started in late fall or winter; and (3) there was no notable harvesting in summer months.

Zooarchaeological analysis of Invertebrate Fauna

Invertebrate fauna were sorted into taxonomic groups and identified to the lowest possible taxon using the comparative collections at the Florida Museum of Natural History, Environmental Archaeology Program. The specimens were counted, weighed and MNI (minimum numbers of individuals) determined. Oysters were the dominant food taxon in sampled material, comprising over 40% of the MNI. In Trench 281 W83 S2, the quick gloss terrestrial snail (*Zonitoides arboreus*) is dominant. However, this is a commensal species attracted to disturbed habitats and not a part of the subsistence economy (Table S1). Table S2 includes collection accession numbers (catalogue numbers), geographic and stratigraphic information for archaeological and modern specimen lots.

Table A. Fauna identified from column samples. Invertebrate fauna identified from column samples 281 W83 S2, 789N801E and 784N811E.

Trench 281 W83 S2							
		**Count**		**MNI**		**WT. (g)**	
**Taxon**	**Common name**	**#**	**%**	**#**	**%**		**%**
Balanus sp.	barnacles	517	10.59	36	1.17	57.92	0.24
Brachyura	crab	402	8.23	-	-	11.86	0.05
Callinectes sp.	blue crab	37	0.76	4	0.13	6.93	0.03
Mollusca	mollusks	-	-	-	-	8110.06	32.97
Geukensia demissa	ribbed mussel	170	3.48	58	1.88	2929.63	11.91
Crassostrea virginica	eastern oyster	1197	24.52	697	22.62	12106.55	49.21
Mercenaria sp.	quahog	24	0.49	4	0.13	477.54	1.94
Tagelus plebius	stout tagelus	137	2.81	65	2.11	634.04	2.58
Gastropoda (marine)	marine snails	3	0.06	-	-	1.01	0.00
Littorina irrorata	marsh periwinkle	296	6.06	268	8.70	251.34	1.02
Triphora nigromaculatus	black-lined triphora	1	0.02	1	0.03	<0.01	-
Urosalpinx sp.	oyster drill	1	0.02	1	0.03	<0.01	-
Collumbellidae	dovesnails	10	0.20	10	0.32	0.07	0.00
Ilyanassa obsoleta	eastern mud snail	9	0.18	9	0.29	2.82	0.01
cf. *Marginella* sp.	marginellas	1	0.02	1	0.03	0.05	0.00
Conidae	cones	1	0.02	1	0.03	0.05	0.00
Boonea impressa	impressed odostome	271	5.55	271	8.79	2.56	0.01
Gastropoda (terrestrial)	terrestrial snails	1	0.02	-	-	0.35	0.00
Haplotrema concavum	gray-footed lancetooth	1	0.02	1	0.03	0.12	0.00
Helicodiscus parallelus	compound coil	291	5.96	291	9.44	0.60	0.00
Polygyridae		98	2.01	-	-	0.26	0.00
Mesodon thyroidus	white-lip globe	3	0.06	3	0.10	0.23	0.00
Polygyra sp.		39	0.80	-	-	0.18	0.00
Polygyra cereolus	southern flatcoil	74	1.52	72	2.34	1.53	0.01
Triodopsis hopeteonensis	magnolia threetooth	5	0.10	5	0.16	0.18	0.00
Gastrocopta contracta	bottleneck snaggletooth	269	5.51	265	8.60	0.38	0.00
Gastrocopta pellucida	slim snaggletooth	17	0.35	17	0.55	0.00	0.00
Euglandina rosea	rosy wolfsnail	2	0.04	2	0.06	0.36	0.00
Strobilops aeneus	bronze pinecone	1	0.02	1	0.03	<0.01	-
Zonitidae		5	0.10	-	-	0.01	0.00

Glyphyalinia umbilicata	Texas glyph	39	0.80	39	1.27	0.02	0.00
Hawaiia miniscula	minute gem	119	2.44	119	3.86	0.11	0.00
Ventridens cerinoideus	wax dome	21	0.43	21	0.68	0.30	0.00
Zonitoides arboreus	quick gloss	820	16.80	820	26.61	3.43	0.01
	**Total**	**4882**	**100**	**3082**	**100**	**24600.50**	**100**

Table A (cont.). Invertebrate fauna identified from column samples from St. Catherines Shell Ring.

789N801E							
		**Count**		**MNI**		**WT. (g)**	
**Taxon**	**Common name**	**#**	**%**	**#**	**%**		**%**
Balanus sp.	barnacles	3665	49.99	73	3.49	106.40	0.29
Brachyura	crabs	376	5.13	-	-	11.74	0.03
Callinectes sp.	swimming crabs	93	1.27	-	-	45.62	0.12
Callinectes sapidus	blue crab	14	0.19	10	0.48	0.39	0.00
Mollusca	mollusks	-	-	-	-	5163.34	13.96
Bivalvia	bivalves	1	0.01	-	-	<0.01	-
Geukensia demissa	ribbed mussel	254	3.46	123	5.88	3336.26	9.02
Crassostrea virginica	eastern oyster	1917	26.15	960	45.87	27996.37	75.70
Mercenaria sp.	quahog	9	0.12	3	0.14	165.79	0.45
Tagelus sp.	tagelus	11	0.15	-	-	1.32	0.00
Tagelus plebius	stout tagelus	55	0.75	27	1.29	136.86	0.37
Gastropoda (marine)	marine snails	4	0.05	-	-	0.33	0.00
Littorina irrorata	marsh periwinkle	37	0.50	14	0.67	12.82	0.03
Triphora nigromaculatus	black-lined triphora	1	0.01	1	0.05	<0.1	-
Columbellidae	dovesnails	2	0.03	2	0.10	<0.1	-
Ilyanassa obsoleta	eastern mudsnail	5	0.07	5	0.24	3.37	0.01
Boonea impressa	impressed odostome	205	2.80	205	9.79	1.33	0.00
Gastropoda (terrestrial)	terrestrial snails	9	0.12	-	-	0.02	0.00
Haplotrema concavum	gray-footed lancetooth	2	0.03	2	0.10	0.28	0.00
Euconulus chersinus	wild hive	1	0.01	1	0.05	<0.01	-
Helicodiscus parallelus	compound coil	125	1.70	125	5.97	0.24	0.00
Polygyra sp.		1	0.01	1	0.05	0.01	0.00
Triodopsis hopetonensis	magnolia threetooth	6	0.08	6	0.29	0.16	0.00
Gastrocopta sp.		1	0.01	-	-	<0.01	-
Gastrocopta contracta	bottleneck snaggletooth	151	2.06	151	7.21	0.31	0.00
Gastrocopta pellucida	slim snaggletooth	7	0.10	7	0.33	<0.01	-
Euglandina rosea	rosy wolfsnail	1	0.01	1	0.05	0.22	0.00
Strobilps aeneus	bronze pinecone	6	0.08	6	0.29	0.01	0.00
Zonitidae		1	0.01	-	-	0.01	-
Glyphyalinia umbilicata	Texas glyph	56	0.76	56	2.68	0.13	0.00
Hawaiia miniscula	minute gem	99	1.35	99	4.73	0.11	0.00
Ventridens cerinoideus	wax dome	12	0.16	10	0.48	0.19	0.00
Zonitoides arboreus	quick gloss	205	2.80	205	9.79	0.70	0.00
	**Total**	**7332**	**100**	**2093**	**100**	**36984.33**	**100**

Table A (cont.). Invertebrate fauna identified from column samples from St. Catherines Shell Ring.

784N811E							
**Taxon**	**Common name**	**#**	**%**	**#**	**%**		**%**
Balanus sp.	barnacles	3008	35.91	134	5.97	212.40	0.78
cf. Brachyura	crabs	2	0.02	-	-	0.76	0.00
Brachyura	crabs	259	3.09	-	-	19.08	0.07
Callinectes sp.	blue crab	47	0.56	3	0.13	18.15	0.07
Mollusca	mollusks	1122.68	13.40	-	-	4610.13	17.02
Bivalvia	bivalves	1	0.01	-	-	0.55	0.00
Geukensia demissa	ribbed mussel	419	5.00	181	8.06	418.16	1.54
Crassostrea virginica	eastern oyster	1932	23.07	971	43.25	21101.96	77.89
Mercenaria spp.	quahogs	30	0.36	-	-	41.61	0.15
Mercenaria sp.	quahog	7	0.08	1	0.04	46.52	0.17
Tagelus sp.	tagelus	43	0.51	-	-	3.64	0.01
Tagelus plebius	stout tagelus	263	3.14	71	3.16	319.14	1.18
Cyrtopleura costata	angelwing	3	0.04	2	0.09	1.83	0.01
Gastropoda (marine)	marine snails	3	0.04	-	-	0.20	0.00
Gastropoda (marine) large	large marine snails	2	0.02	1	0.04	0.20	0.00
Littorina sp.	periwinkle	14	0.17	-	-	0.19	0.00
Littorina irrorata	marsh periwinkle	535	6.39	197	8.78	285.21	1.05
Crepidula sp.	slippersnail	1	0.01	1	0.04	0.01	0.00
Collumbellidae	dovesnails	4	0.05	4	0.18	0.01	0.00
Illyanassa obsoleta	eastern mudsnail	6	0.07	6	0.27	2.63	0.01
Boonea impressa	impressed odostome	285	3.40	285	12.69	8.33	0.03
Haplotrema concavum	gray-footed lancetooth	4	0.05	4	0.18	0.02	0.00
Euconulus chersinus	wild hive	1	0.01	1	0.04	<0.01	0.00
Oligyra orbiculata	globular drop	1	0.01	1	0.04	0.09	0.00
Helicodiscus parallelus	compound coil	128	1.53	128	5.70	0.25	0.00
Polygyridae		1	0.01	-	-	0.05	0.00
Lobosculum pustula	grooved liptooth	5	0.06	5	0.22	0.02	0.00
Polygyra cereolus	southern flatcoil	1	0.01	1	0.04	0.02	0.00
Triodopsis hopetonensis	magnolia threetooth	6	0.07	6	0.27	0.28	0.00
Gastrocopta contracta	bottleneck snaggletooth	54	0.64	54	2.41	0.04	0.00
Gastrocopta pellucida	slim snaggletooth	4	0.05	4	0.18	<0.01	0.00
Euglandina rosea	rosy wolfsnail	1	0.01	1	0.04	0.20	0.00
Strobilops aeneus	bronze pinecone	1	0.01	1	0.04	<0.01	0.00
Glyphyalinia umbilicata	Texas glyph	19	0.23	19	0.85	0.03	0.00
Hawaiia miniscula	minute gem	17	0.20	17	0.76	<0.01	0.00
Ventridens cerinoideus	wax dome	18	0.21	18	0.80	0.32	0.00
Zonitoides arboreus	quick gloss	128	1.53	128	5.70	0.59	0.00
	**Total**	**8375.68**	**100**	**2245**	**100**	**27092.61**	**100**

Table B. Catalog numbers and repository location. Accessioned lots of modern and archaeological *Boonea impressa* samples reported in this study.

Modern *Boonea impressa* samples
St. Catherines Island, GA
Cemetery Road Marsh (31°37’47”N 81°9’16”W)

Collection Date	Screen size	Specimen Count	Catalog number
July 2006	1.168mm	224	47052-001
July 2006	250µm	7	47053-001
August 2006	1.168mm	9	47054-001
August 2006	250µm	25	47055-001
September 2006	1.168mm	75	47056-001
September 2006	250µm	38	47057-001
October 2006	250µm	2	47058-001
November 2006	1.168mm	39	47059-001
November 2006	250µm	5	47060-001
December 2006	1.168mm	38	47061-001
December 2006	250µm	1	47062-001
January 2007	1.168mm	228	47063-001
February 2007	1.168mm	32	47064-001
March 2007	1.168mm	25	47065-001
April 2007	1.168mm	65	47066-001
May 2007	1.168mm	6	47067-001
June 2007	1.168mm	2	47068-001
June 2007	250µm	129	47069-001
July 2007	250µm	743	47070-001
August 2007	250µm	25	47071-001
September 2007	1.168mm	890	47072-001
September 2007	250µm	468	47073-001
October 2007	1.168mm	220	47074-001
October 2007	250µm	2	47075-001
November 2007	1.168mm	196	47076-001
November 2007	250µm	18	47077-001
December 2007	1.168mm	198	47078-001
December 2007	250µm	3	47079-001
January 2008	1.168mm	26	47080-001
January 2008	250µm	1	47081-001
February 2008	1.168mm	75	47082-001
February 2008	250µm	1	47083-001
March 2008	1.168mm	73	47084-001
March 2008	250µm	3	47085-001
April 2008	1.168mm	37	47086-001
April 2008	250µm	1	47087-001
May 2008	1.168mm	64	47088-001
May 2008	250µm	92	47089-001
June 2008	1.168mm	1	47090-001
June 2008	250µm	232	47091-001

Table B (cont.). Accessioned lots of modern and archaeological *Boonea impressa* samples reported in this study.

Site Location: St. Catherines Island, GA

Site: St. Catherines Shell Ring

Unit: Trench 281 W83S2

Boonea impressa catalog numbers

Current collection Location: Environmental Archaeology Laboratory, Florida Museum of Natural History, Gainesville Florida, USA

Permanent Collection Location: Laboratory of Archaeology, University of Athens Georgia, USA

Unit	Depth	Screen	Taxon	Catalog number
W83S2	3.0-2.9	1/16	*Boonea impressa*	1601-001
W83S2	2.9-2.75	1/8	*Boonea impressa*	1602-001
W83S2	2.9-2.75	1/16	*Boonea impressa*	1602-002
W83S2	2.75-2.7	1/8	*Boonea impressa*	1603-001
W83S2	2.75-2.7	1/16	*Boonea impressa*	1603-002
W83S2	2.7-2.6	1/8	*Boonea impressa*	1652-001
W83S2	2.7-2.6	1/16	*Boonea impressa*	1652-002
W83S2	2.6-2.5	1/8	*Boonea impressa*	1653-001
W83S2	2.6-2.5	1/16	*Boonea impressa*	1653-002
W83S2	2.5-2.4	1/8	*Boonea impressa*	1745-001
W83S2	2.5-2.4	1/16	*Boonea impressa*	1745-002
W83S2	2.36-2.30	1/16 dry	*Boonea impressa*	1749-001
W83S2	2.40-2.28	1/8	*Boonea impressa*	1747-001
W83S2	2.40-2.28	1/16	*Boonea impressa*	1747-002
789N801E	3.0-2.9	1/16	*Boonea impressa*	1931-001
789N801E	2.9-2.8	1/16	*Boonea impressa*	1932-001
789N801E	2.9-2.8	1/8	*Boonea impressa*	1932-001
789N801E	2.9-2.8	1/16	*Boonea impressa*	1932-002
789N801E	2.8-2.7	1/8	*Boonea impressa*	1924-001
789N801E	2.8-2.7	1/16	*Boonea impressa*	1924-002
789N801E	2.7-2.6	1/8	*Boonea impressa*	1928-001
789N801E	2.7-2.6	1/16	*Boonea impressa*	1928-002
789N801E	2.6-2.5	1/8	*Boonea impressa*	1925-001
789N801E	2.6-2.5	1/16	*Boonea impressa*	1925-002
789N801E	2.5-2.4	1/8	*Boonea impressa*	1923-001
789N801E	2.5-2.4	1/16	*Boonea impressa*	1923-002
789N801E	2.4-2.3	1/16	*Boonea impressa*	1927-001
789N801E	2.3-2.2	1/8	*Boonea impressa*	1926-001
789N801E	2.3-2.2	1/16	*Boonea impressa*	1926-002
789N801E	2.2-2.1	1/16	*Boonea impressa*	1929-001
784N811E	2.9-2.8	1/4	*Boonea impressa*	1944-001
784N811E	2.9-2.8	1/8	*Boonea impressa*	1944-002
784N811E	2.9-2.8	1/16	*Boonea impressa*	1944-003
784N811E	2.8-2.7	1/8	*Boonea impressa*	1930-001
784N811E	2.8-2.7	1/16	*Boonea impressa*	1930-002
784N811E	2.7-2.6	1/4	*Boonea impressa*	1940-001
784N811E	2.7-2.6	1/8	*Boonea impressa*	1940-002
784N811E	2.7-2.6	1/16	*Boonea impressa*	1940-003
784N811E	2.6-2.5	1/8	*Boonea impressa*	1941-001
784N811E	2.6-2.5	1/16	*Boonea impressa*	1941-002
784N811E	2.5-2.4	1/4	*Boonea impressa*	1946-001
784N811E	2.5-2.4	1/8	*Boonea impressa*	1946-002
784N811E	2.5-2.4	1/16	*Boonea impressa*	1946-003
784N811E	2.4-2.3	1/4	*Boonea impressa*	1942-001
784N811E	2.4-2.3	1/8	*Boonea impressa*	1942-002

Table B (cont.). Accessioned lots of modern and archaeological *Boonea impressa* samples reported in this study.

Unit	Depth	Screen	Taxon	Catalog number
784N811E	2.4-2.3	1/16	*Boonea impressa*	1942-003
784N811E	2.3-2.2	1/4	*Boonea impressa*	1945-001
784N811E	2.3-2.2	1/8	*Boonea impressa*	1945-002
784N811E	2.3-2.2	1/16	*Boonea impressa*	1945-003
