# Supplementary figures and images for: Seasonal oyster harvesting recorded in a Late Archaic period shell ring
Source: PLoS One. 2019 Nov 20;14(11):e0224666. doi: 10.1371/journal.pone.0224666 (PMC6867601; doi:10.1371/journal.pone.0224666)

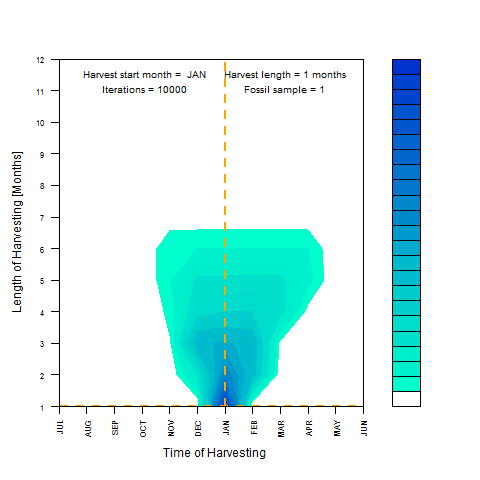

Supplement: S1 Movie — Animation showing a sequence of 133 null models depicted in Fig 9. (GIF) [file pone.0224666.s002.gif]

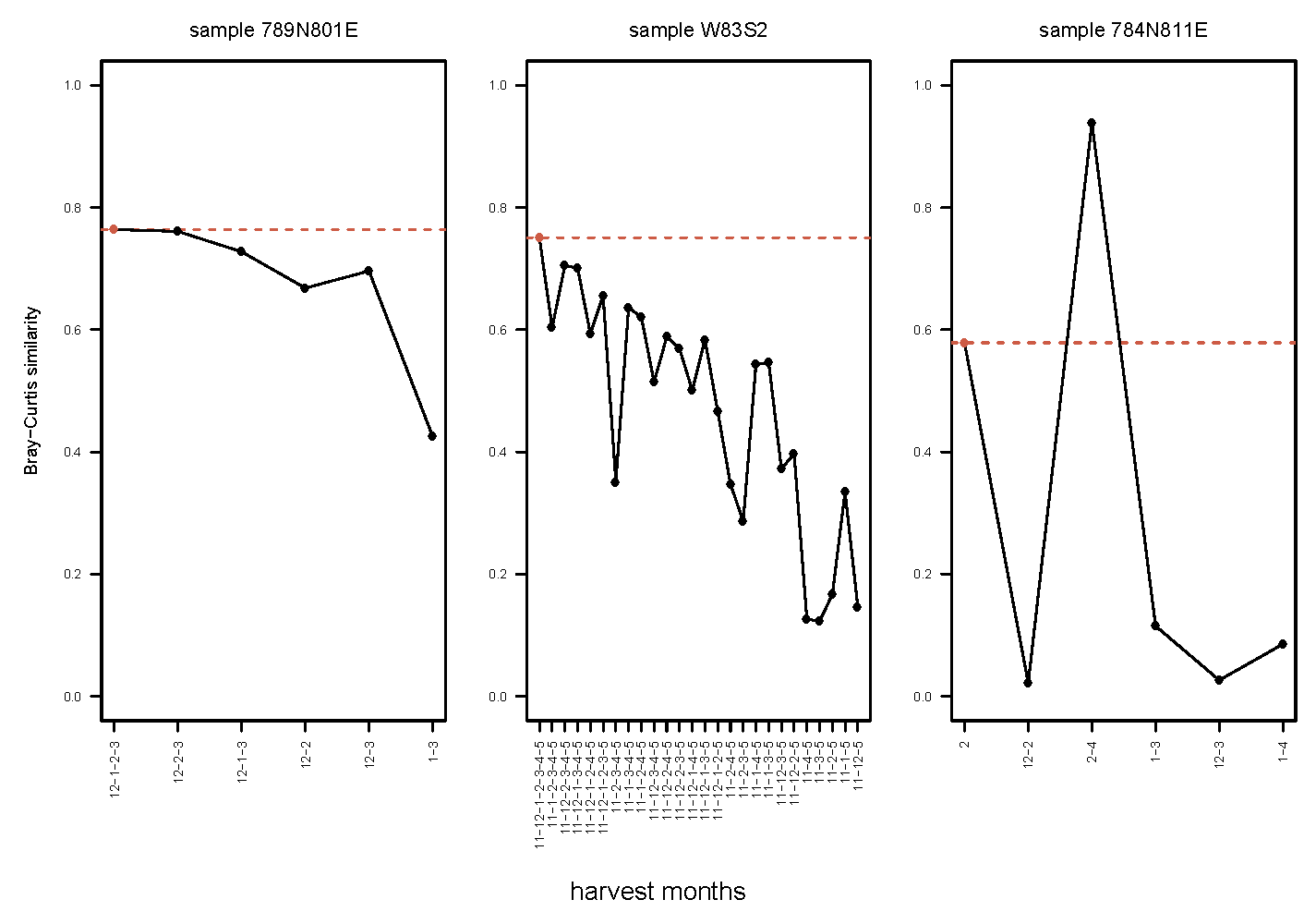

Supplement: S2 Fig — Difference between monthly size-frequency distributions of live-collected specimens of Boonea impressa plotted as a function of sample size (n) of the smaller of the two compared monthly samples. Each data point represents a pairwise comparison for the same month from different years (e.g., March 2007 vs. March 2008). A. Pairwise differences in median shell length between the two compared years for a given month. B. Kolmogorov-Smirnov D statistic measuring overall difference in the shape of the two compared size-frequency distributions. (TIF) [file pone.0224666.s004.tif]
